# Amnion signals are essential for mesoderm formation in primates

**DOI:** 10.1038/s41467-021-25186-2

**Published:** 2021-08-26

**Authors:** Ran Yang, Alexander Goedel, Yu Kang, Chenyang Si, Chu Chu, Yi Zheng, Zhenzhen Chen, Peter J. Gruber, Yao Xiao, Chikai Zhou, Nevin Witman, Elif Eroglu, Chuen-Yan Leung, Yongchang Chen, Jianping Fu, Weizhi Ji, Fredrik Lanner, Yuyu Niu, Kenneth R. Chien

**Affiliations:** 1 Department of Cell and Molecular Biology, Karolinska Institutet, Stockholm, Sweden; 2 Yunnan Key Laboratory of Primate Biomedical Research, Institute of Primate Translational Medicine, Kunming University of Science and Technology, Kunming, Yunnan China; 3 Department of Mechanical Engineering, University of Michigan, Ann Arbor, MI USA; 4 Department of Surgery, Yale University, New Haven, CT USA; 5 Department of Cell and Developmental Biology, University of Michigan Medical School, Ann Arbor, MI USA; 6 Department of Biomedical Engineering, University of Michigan, Ann Arbor, MI USA; 7 Department of Clinical Sciences, Intervention and Technology, Karolinska Institutet, Stockholm, Sweden; 8 Division of Obstetrics and Gynecology, Karolinska Universitetssjukhuset, Stockholm, Sweden; 9 Ming Wai Lau Center for Reparative Medicine, Stockholm Node, Karolinska Institutet, Stockholm, Sweden; 10 Faculty of Life Science and Technology, Kunming University of Science and Technology, Kunming, Yunnan China

**Keywords:** Embryology, Mesoderm, Stem-cell differentiation

## Abstract

Embryonic development is largely conserved among mammals. However, certain genes show divergent functions. By generating a transcriptional atlas containing >30,000 cells from post-implantation non-human primate embryos, we uncover that *ISL1*, a gene with a well-established role in cardiogenesis, controls a gene regulatory network in primate amnion. CRISPR/Cas9-targeting of *ISL1* results in non-human primate embryos which do not yield viable offspring, demonstrating that *ISL1* is critically required in primate embryogenesis. On a cellular level, mutant *ISL1* embryos display a failure in mesoderm formation due to reduced BMP4 signaling from the amnion. Via loss of function and rescue studies in human embryonic stem cells we confirm a similar role of *ISL1* in human in vitro derived amnion. This study highlights the importance of the amnion as a signaling center during primate mesoderm formation and demonstrates the potential of in vitro primate model systems to dissect the genetics of early human embryonic development.

## Abstract (web summary)

Human and murine embryonic development has disparities, highlighting the need for primate systems. Here, the authors construct a post-implantation transcriptional atlas from non-human primate embryos and show ISL1 controls a gene regulatory network in the amnion required for mesoderm formation.

## Introduction

Studies in genetically modified model organisms, in particular the mouse, have allowed us to investigate mammalian embryonic development in astonishing detail and have laid the foundations to understanding human development. However, in certain cases, the phenotype observed in knockout mouse models differs from observations in human cohorts. The LIM-domain transcription factor *ISL1* has a well-established role in mammalian cardiac development and is expressed in multipotent cardiovascular progenitor cells in mice^1–3^ and humans^4,5^. In line with this, *Isl1* loss-of-function mice have severe cardiac defects leading to embryonic lethality at embryonic day 10.5 (E10.5)^6,7^. Despite its established role in heart development, loss-of-function variants in the *ISL1* locus have rarely been associated with cardiac defects in humans and are underrepresented in large human cohorts of congenital heart malformations like the Pediatric Cardiac Genomics Consortium (PCGC)^8,9^. In detail, among the 23,000 alleles reported in the PCGC cohort, 112 *ISL1* variants have been identified, none of which were damaging de novo mutations^8,9^. Based on this low frequency of damaging *ISL1* variants, we hypothesize that *ISL1* has an alternative, essential requirement during early primate embryogenesis.

Studies of in vitro cultured human embryos have shown that *ISL1* is not expressed in the preimplantation blastocyst^10^. One of the key steps during mammalian development following implantation is the formation of the three primary germ layers. This occurs in a complex process termed gastrulation where cells from the columnar-shaped epiblast undergo epithelial-to-mesenchymal transition and move ventrally and anteriorly to form the mesodermal cells^11–13^. It is believed that improper gastrulation occurs frequently in human embryos and accounts for a significant proportion of early miscarriages in the human population.

The tight regulatory network governing this process has been well studied during murine embryonic development^11,14^, but is largely elusive in humans. Recently, two publications on cynomolgus embryogenesis^15,16^ and one publication on human embryogenesis^17^ have created a framework of this developmental time window in primates and characterized the major cell populations involved in gastrulation. However, their interplay and the transcriptional networks guiding this essential step remain unknown.

Here, we created a high-resolution map of the peri-gastrulation development of non-human primate (NHP) embryogenesis, which we made accessible through an online resource reachable at http://www.nhp-embryo.net. We identify an *ISL1*-dependent gene regulatory network (GRN) that is specifically active in the amnion. Disturbance of this network in NHP embryos by CRIPSR/Cas9-mediated gene editing of *ISL1* led to embryonic lethality due to significant downregulation of bone morphogenetic protein 4 (BMP4) signaling from the amnion and subsequent failure to form mesoderm. We confirmed these findings in a microfluidic-based embryonic sac model of amnion–epiblast interactions using *ISL1*-null human embryonic stem cells (hESCs) suggesting that these findings also apply to humans. Taken together, this study demonstrates a primate-specific role of *ISL1* in early embryogenesis and shows that signals from the amnion are indispensable for mesoderm formation in primate embryos.

## Results

### Loss of *ISL1* leads to embryonic lethality

To assess whether *ISL1* plays a functional role in primate embryogenesis, we generated *ISL1* mutant NHP embryos through one-cell stage CRISPR/Cas9 injections with two guide RNAs (gRNAs) designed to create a long deletion in the *ISL1* locus (Supplementary Fig. 1a). PCR-based genotyping of the mutant embryos showed 100% editing efficiency (Supplementary Fig. 1a). However, within each embryo, we observed the presence of indels of different sizes in the targeted region of the *ISL1* locus (Supplementary Fig. 1a).

Most of the indels (81–98%) were large deletions causing a frameshift. With low frequency (2–19%), we observed in-frame 9 bp deletions resulting in a loss of the first three amino acids from the N terminus (Supplementary Fig. 1a). Single-cell genotyping confirmed the mosaic pattern on the cellular level with most of the cells (>95%) carrying frameshift mutations in the *ISL1* locus (Supplementary Fig. 1b). We did not find any alterations in selected off-targets from the in silico prediction^18^ (Supplementary Fig. 1c).

After transfer, the pregnancy rate per NHP surrogate mother as assessed by ultrasound imaging from 4 weeks of gestation was 0% with *ISL1* targeted embryos as compared to 58.3% with wild-type embryos (Supplementary Fig. 1d, e). Transfer of embryos that were targeted with an injection of only a single gRNA, leading to a slightly lower mutation rate, resulted in a pregnancy rate of 28.6% (Supplementary Fig. 1e). Strikingly, genotyping of all four fetuses from this experiment showed an unmodified *ISL1* locus on both alleles, confirming the suspected early requirement of *ISL1* for proper embryonic development.

### Single-cell map of postimplantation NHP embryos

**Fig. 1:**
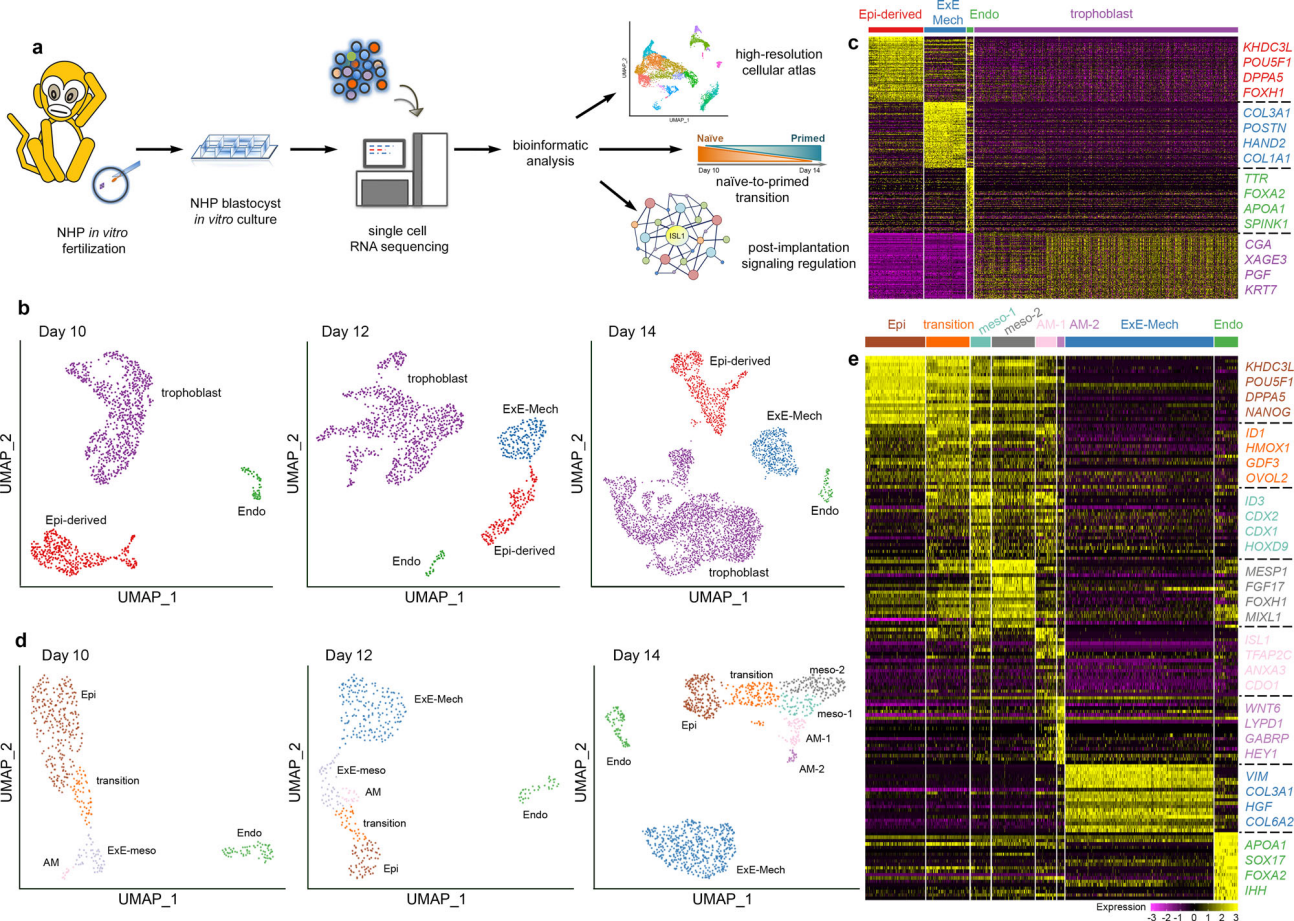
High-resolution transcriptomic map of peri-gastrulation events in wild-type in vitro cultured NHP embryos. See also Supplementary Figs. 1–5 and Supplementary Data 1–3. **a** Scheme of the workflow. **b** UMAP plot of all cells from the in vitro cultured embryos at the different time points (Days 10, 12, and 14) colored by cell type. **c** Heatmap showing the scaled expression at Days 14 of 100 differentially expressed genes (DEGs) for each cell type identified in (**b**) selected by false discovery rate. **d** UMAP plot of cells from the in vitro cultured embryos at the different time points (Days 10, 12, and 14) mapping to the Epi and its derivatives, Endo and ExE-Mech colored by cell type. **e** Heatmap showing the scaled expression at Days 14 of 20 DEGs for each cell type identified in (**d**) selected by false discovery rate. Epi-derived epiblast and epiblast-derived cells, ExE-Mech extraembryonic mesenchyme, Endo endoderm, Epi epiblast, ExE-meso extraembryonic mesoderm, AM amnion, AM-1 amnion 1, AM-2 amnion 2, meso-1 mesoderm 1, meso-2 mesoderm 2.

To map the expression of *ISL1* in the early embryo, we created a high-resolution transcriptomic atlas by single-cell RNA (scRNA) sequencing of 12 in vitro cultured cynomolgus macaque embryos at three different time points (Day 10, Day 12, and Day 14) (Fig. 1a). 7194 cells passing quality control (Supplementary Fig. 2) were embedded for each day separately in low-dimensional space (Fig. 1b and Supplementary Fig. 3a). In line with previous results^15,16^, the cells grouped into four main cell types, namely trophoblast, endoderm, and epiblast with its derivatives and extraembryonic mesenchyme (ExE-Mech) (Fig. 1b, c and Supplementary Data 1). Integration of our dataset with a published scRNA-sequencing dataset of in vivo cynomolgus embryos^19^ (Supplementary Fig. 3b, c) revealed a striking difference in the transcriptomic profile between early (Day 10 + E08/E09) and late (Day 12/Day 14 + E13/E14) epiblast, reflecting the transition from a naive to a primed state, which has been suggested before to happen during this time window^20^. Indeed, a published gene signature of naive hESCs^21^ was highly enriched in the early peri-implantation epiblast at Day 10, while genes belonging to the primed hESCs signature were enriched in the late epiblast at Days 12 and 14 (Supplementary Fig. 3c, d). Aligning cells from the early and late peri-implantation epiblast in pseudotime disclosed a set of differentially regulated genes that formed two distinct clusters based on their expression dynamics (Supplementary Fig. 3e). Genes previously associated with a naive state^20–22^, including *DNMT3L*, *KHDC1L*, *NLRP7*, *OOEP*, and *DPP4* were significantly downregulated over pseudotime (Supplementary Fig. 3f and Supplementary Data 2). In contrast, genes associated with a primed state^22–24^, including *CD24*, *CRABP2*, *SFRP1*, *USP44*, and *VCAN*, showed strong upregulation (Supplementary Fig. 3f and Supplementary Data 2). This expression pattern was observed in cells from our dataset as well as in cells from the in vivo dataset, suggesting that the naive to primed transition happening in vivo can faithfully be recapitulated in in vitro cultured embryos.

### Epiblast-derived cell populations in the NHP embryo

Taking advantage of our high-resolution scRNA map, we next investigated the appearance of epiblast-derived cell populations (Fig. 1d, e, Supplementary Fig. 4a, and Supplementary Data 1). Embryos at Day 10 consisted of a relatively large-cell population expressing genes typical of a mesodermal signature such as *MIXL1* and *MESP1* (Supplementary Fig. 4b), which were previously annotated as early gastrulating cells^15^. However, in addition to their mesodermal signature, these cells show high expression of the transcription factor *ETS1* and the cell adhesion protein *PODXL* (Supplementary Fig. 4c), which mark extraembryonic mesoderm in mice^25^, while showing low levels of expression of the receptor tyrosine kinase *EPHA4* or the transcription factor *ZIC3* (Supplementary Fig. 4d) expressed preferentially by murine embryonic mesoderm^25^. This suggests that the epiblast-derived mesodermal-like cells present in Day 10 embryos are likely extraembryonic mesoderm, which seem to appear prior to gastrulation.

ExE-Mech, a cell population that contributes to a number of extraembryonic tissues in primates^19,26–28^, was first present in Day 12 embryos (Fig. 1d). The close proximity of extraembryonic mesoderm and mesenchyme in the Uniform Manifold Approximation and Projection (UMAP) plot (Fig. 1d), as well as a shared expression of genes such as *PODXL* and *ETS1* (Supplementary Fig. 4e), advocate that extraembryonic mesoderm develops into ExE-Mech. The large increase in cell number in the ExE-Mech over a few days and shared expression of genes with cells in the endoderm including *PITX1*, *LUM*, and *NID2* at Day 12 (Supplementary Fig. 4f) as well as Day 14 (Supplementary Fig. 4g) could indicate that this cell population gets additional contributions from the endoderm as previously suggested^29^.

In Day 14 embryos, we identified two clusters of embryonic mesoderm cells (Fig. 1d, e). Cells in cluster mesoderm 1 (meso-1) preferentially expressed members of the caudal gene family *CDX1*, *CDX2*, and *CDX4*, while cells in the cluster mesoderm 2 (meso-2) were expressing relatively higher levels of *MESP1* and *GSC* (Supplementary Fig. 4h, i). Several mesodermal marker genes such as *TBXT*, *MESP1*, and *MIXL1* were exclusively expressed in cells of the embryonic mesoderm clusters, while others including *FOXF1*, *PDGFRA*, and *HAND1* showed shared expression with the ExE-Mech (Supplementary Fig. 4i–k). Transition cells found in Day 10, 12, and 14 embryos shared expression of genes with cells in the mesodermal as well as epiblast clusters most likely resembling epiblast cells in the process of differentiation towards mesoderm (Fig. 1d, e).

Cells expressing amnion marker genes such as *WNT6*^15,30^ alongside with *ISL1* were found as early as Day 10, and also at Day 12 (Fig. 1d and Supplementary Fig. 5a, b). In the Day 14 embryo, two clusters showed a gene expression profile consistent with amnion cells^15,30^ and were labeled as AM-1 and AM-2 (Fig. 1d, e and Supplementary Fig. 5c). Of the identified marker genes, *WNT6*, *GABRP*, and *ISL1* showed specific expression in amnion cells, while *HEY1* was also expressed in subpopulations of the trophoblast (Supplementary Fig. 5d). Across all time points, amnion cells showed no expression of the pluripotency marker *SOX2*^22^ (Supplementary Fig. 5a–c). Some amnion-specific genes, in particular *WNT6*, showed restricted expression in amnion cells from early stages onward, while others such as *GABRP* were only expressed at Day 14 (Supplementary Fig. 5a–c). Notably, the latter is a specific marker for the AM-2 population, which seems to be absent from the early embryo.

To identify the defining features of the two different amnion populations, differential gene expression analysis followed by STRING network analysis between AM-1 and AM-2 was performed. Cells in AM-1 expressed higher levels of genes associated with pluripotency such as *DPPA5* and *KHDC3L*, several HOX genes such as *HOXD4*, *HOXA5*, *HOXB6*, and *HOXB9*, as well as members of the caudal gene family such as *CDX1* and *CDX2* (Supplementary Data 3). Of the 72 genes significantly upregulated in AM-2, 42 formed a tightly interconnected network (protein–protein interaction (PPI) enrichment *p* value: <1.0e − 16) (Supplementary Fig. 5e). Among the most significantly enriched Gene Ontology (GO) terms was epithelium development (GO:0060429) as well as epithelial cell differentiation (GO:300855), suggesting that AM-2 represents amniotic epithelium (Supplementary Fig. 5f).

**Fig. 2:**
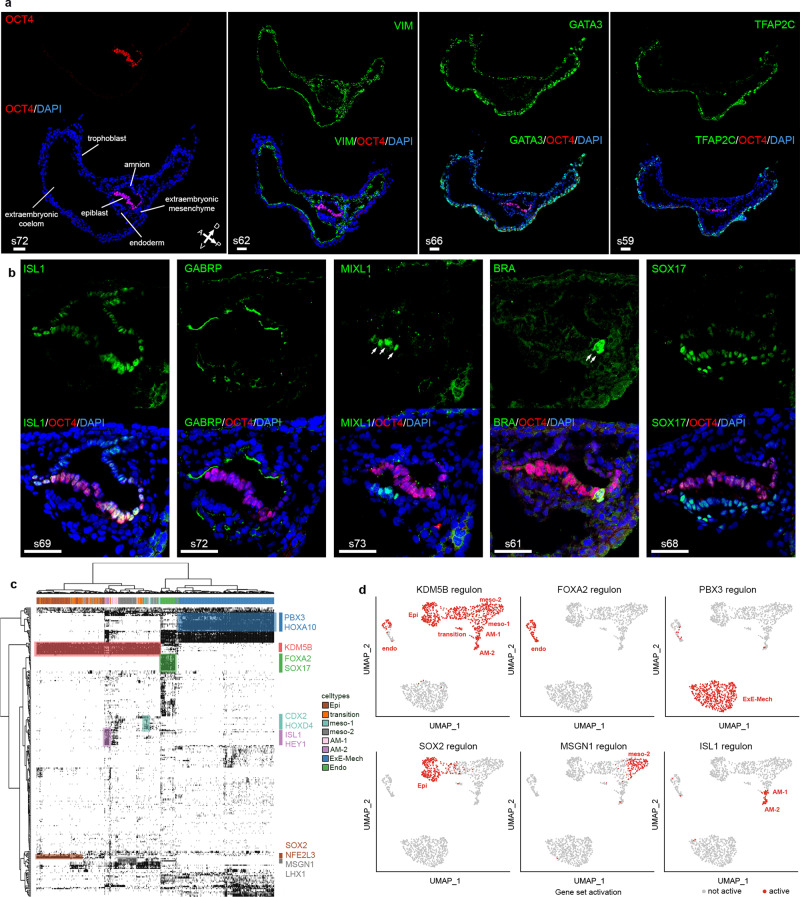
Immunofluorescent staining and gene regulatory networks in wild-type in vitro cultured NHP embryos. See also Supplementary Fig. 6 and Supplementary Data 4. **a**, **b** immunofluorescent staining of major cell types at Day 14 identified in the scRNA sequencing. *n* = 2 wild-type embryos **a** OCT4, VIM, GATA3, TFAP2C; **b** ISL1, GABRP, MIXL1, BRA, and SOX17. Scale bar = 50 µm. White numbers indicate section numbers and arrows indicate the signal of interest. **c** Binary activity matrix of regulons identified at Day 14 by gene regulatory network inference active in at least 1% of the cells clustered unsupervised. Selected master regulators are depicted in the color corresponding to the cell type they show activity in. **d** Binarized gene set activity of the selected regulons at Day 14 in the different cell types depicted on the UMAP plot from Fig. 1d. Epi epiblast, Endo endoderm, ExE-Mech extraembryonic mesenchyme, AM-1 amnion 1, AM-2 amnion 2, meso-1 mesoderm 1, meso-2 mesoderm 2, D dorsal, V ventral, A anterior, P posterior.

Immunofluorescent imaging of sectioned in vitro cultured embryos at Day 14 confirmed the presence of the different cell populations identified in the scRNA-sequencing analysis (Fig. 2a, b). In particular, we observed specific staining for ISL1 in amniotic cells overlying the epiblast staining positive for OCT4 (*POU5F1*) as well as in cells directly adjacent to it (Fig. 2b). Staining for GABRP, which is a marker for the AM-2 population, showed specific labeling of the luminal side of the amniotic epithelium (Fig. 2b). The ISL1-positive cells that are located closer to the embryonic disc lacking epithelial morphology stained negative for GABRP, suggesting that these cells correspond to the AM-1 population (Fig. 2b). Endoderm showed a strong signal for SOX17 (Fig. 2), while trophoblast stained positive for GATA3 (Fig. 2a). Brachyury (BRA, *TBXT*) as well as MIXL1-positive cells were located in close proximity to the embryonic disc (Fig. 2b), suggesting that the mesodermal cells identified in the scRNA-sequencing data at Day 14 are indeed emerging embryonic mesoderm during gastrulation. ExE-Mech, staining positive for Vimentin (*VIM*) was clustering around the embryonic disc, in particular in the region that will most likely develop into the connecting stalk in later stages, but was also lining the entire trophoblast (Fig. 2a).

Cell type specification is under the control of transcription factors that bind to *cis*-regulatory regions, forming GRNs^31^. GRN analysis using SCENIC (single-cell regulatory network inference and clustering)^32^ followed by binarization of the network activity based on the distribution of the area under the curve (AUC) values revealed sets of GRNs specifically active in these populations at the different time points (Fig. 2c, d, Supplementary Fig. 6, and Supplementary Data 4). At Day 14, the histone demethylase *KDM5B*, creating bivalent histone marks during development^9^, was identified to control a GRN active in epiblast and all its derivatives, while the pluripotency factor *SOX2*^24^ controlled a network specifically active in the epiblast (Fig. 2c, d). One of the most active GRNs in amniotic cells was an ISL1-dependent network (Fig. 2d), signifying that *ISL1* is not only a specific marker of the amniotic cell population in primates, but could be functionally important. This finding is in sharp contrast to the mouse, where *Isl1* is first expressed in cardiac progenitor cells of the lateral plate mesoderm, but absent from the early embryo before E7.0^7,33^.

### *ISL1* mutant embryos fail to form mesoderm

**Fig. 3:**
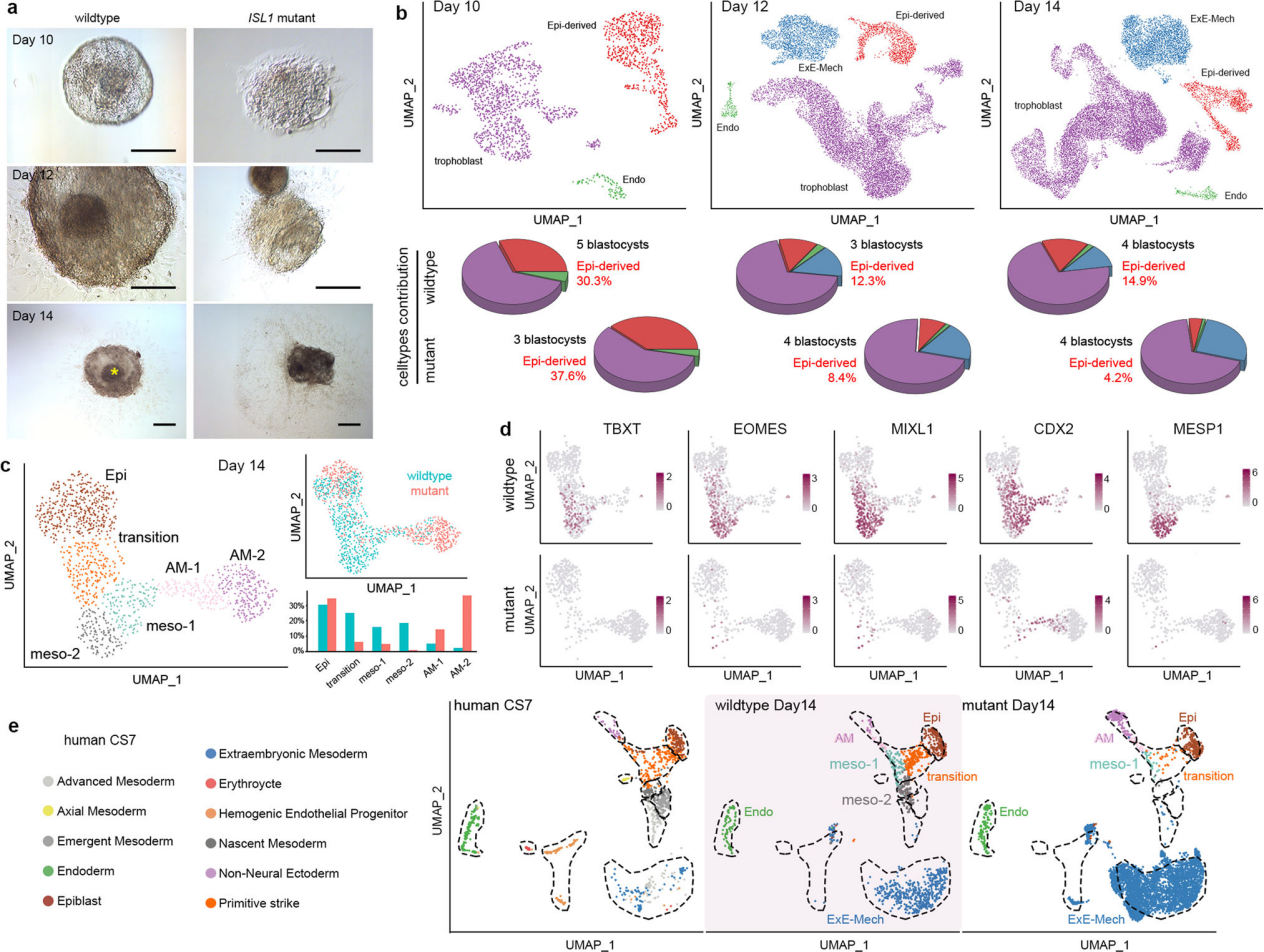
*ISL1* mutants fail to form mesoderm. See also Supplementary Fig. 7. **a** The morphology of wild-type (left) and *ISL1* mutant (right) NHP embryos at Days 10, 12, and 14. Scale bar, 200 µm. Asterisk indicates the embryonic disk. *n* = 15 wild-type and 14 mutant embryos. **b** UMAP plot of all cells from the integrated dataset of wild-type and mutant embryos at the different time points (Days 10, 12, and 14) colored by cell type. Pie charts indicate the relative contribution of cell types. **c** UMAP plot of cells mapping to the epiblast and its derivatives at Day 14 and the relative contribution of cells to the various cell types in wild-type (blue) and mutant (red) embryos. *n* = 4 wild-type and 4 mutant embryos collected in two batches. **d** Expression of mesodermal marker genes in cells from the epiblast and its derivatives of Day 14 wild-type (top) and mutant (bottom) embryos. **e** UMAP plot of the integrated dataset of all cells excluding trophoblast from the in vitro cultured embryos at Day 14 with cells from a human Carnegie stage 7 (CS7) in vivo embryo colored by cell types. Epi-derived epiblast and epiblast-derived cells, Epi epiblast, Endo endoderm, ExE-Mech extraembryonic mesenchyme, AM-1 amnion 1, AM-2 amnion 2, meso-1 mesoderm 1, meso-2 mesoderm 2, wt wild type, mt mutant.

To investigate the functional role of this ISL1-dependent GRN in postimplantation development, the *ISL1* mutant embryos were cultured in vitro. Mutant embryos developed normally up to Day 10 (Fig. 3a and Supplementary Fig. 7a, b). After Day 12, they progressively lost structure, and at Day 14, the embryonic disk was no longer distinguishable (Fig. 3a and Supplementary Fig. 7b). This was reflected in the integrated analysis of 26,136 cells passing quality control from Day 10, 12 and 14 mutant embryos (Supplementary Fig. 2) with the wild-type dataset by a progressive loss of cells in the Epi-derived cluster while the other lineages were preserved (Fig. 3b and Supplementary Fig. 7c). Subclustering of the Epi-derived cells at Day 14 showed a drastic reduction of cells in the mesodermal clusters in the mutant embryos accompanied by an overrepresentation of amniotic cells (Fig. 3c and Supplementary Fig. 7d). In line with this, the number of cells expressing the mesoderm markers *TBXT*, *EOMES*, *MIXL1*, *CDX2*, and *MESP1*^34–36^ was reduced in the mutant embryos among epiblast derivatives (Fig. 3d) as well as across the whole dataset (Supplementary Fig. 7e).

Integration of this dataset with scRNA-sequencing data of an in vivo human Carnegie stage 7 embryo^37^ showed that the mesodermal cell populations underrepresented in the mutant embryos match the primitive streak and nascent mesoderm clusters of the human embryo (Fig. 3e). This strongly supports the conclusion that mutant primate embryos fail to form mesoderm.

**Fig. 4:**
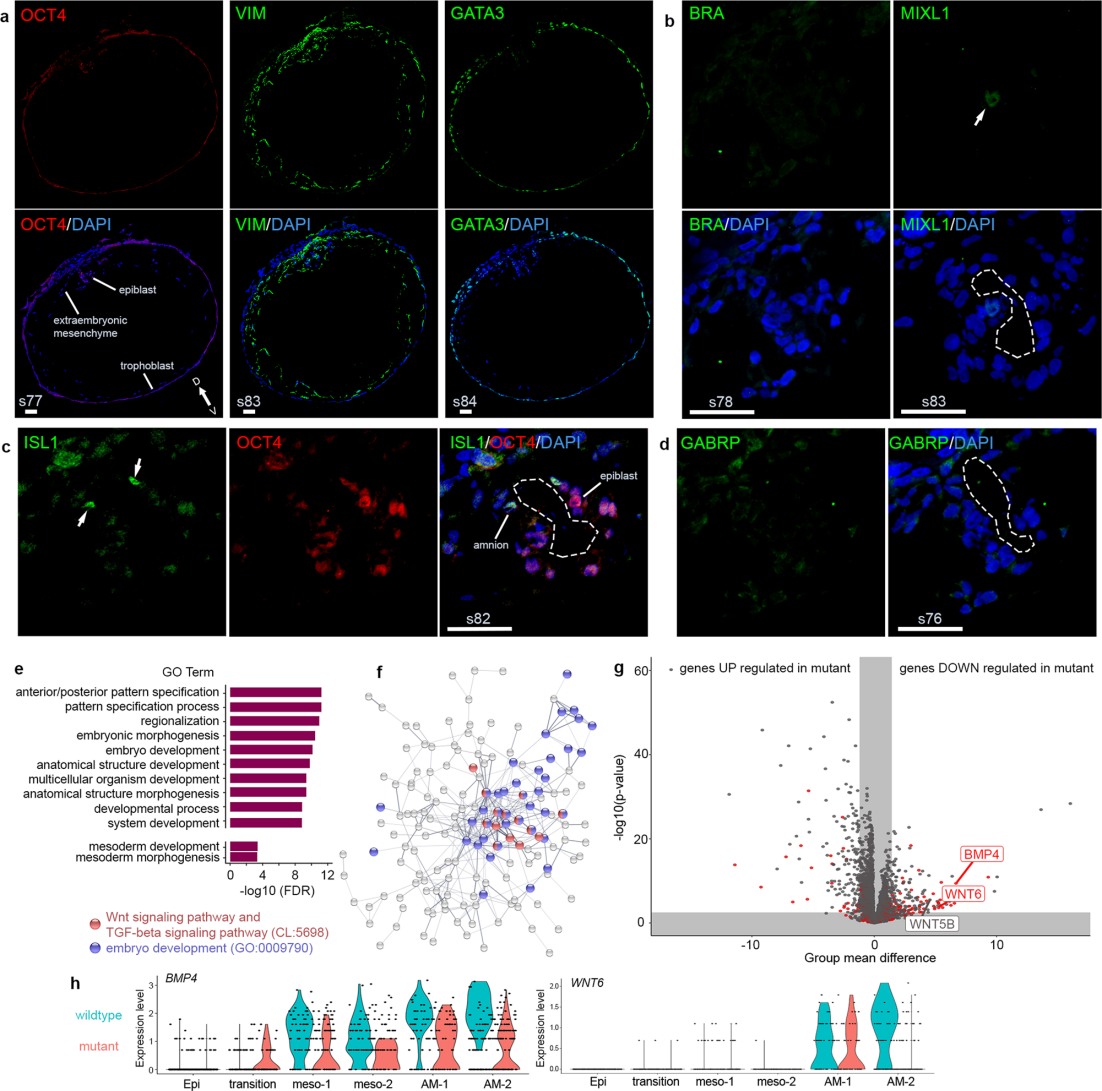
Loss of ISL1 impairs amnion signals essential for mesoderm formation. See also Supplementary Fig. 8 and Supplementary Data 5. **a**–**d** Immunofluorescent staining of major cell types on sections of *ISL1* mutant embryos at Day 14. *n* = 3 mutant embryos. **a** OCT4, VIM, and GATA3; **b** BRA and MIXL1; **c** ISL1; **d** GABRP. Scale bar 50 µm. Numbers represent section numbers. Arrows indicate signals of interest and dashed lines mark the amniotic cavity. **e** GO categories enriched among genes significantly downregulated in cells of the mesodermal clusters in mutant embryos ordered by false discovery rate (FDR). *n* = 4 wild-type and 4 mutant embryos collected in two batches. **f** STRING network of all significantly downregulated genes in cells of the mesodermal clusters in the mutant embryos. Nodes not connected to the main network have been removed. Nodes belonging to the GO category embryonic development colored in blue; nodes belonging to the STRING network cluster Wnt signaling pathway, and TGF-beta signaling pathway colored in red. **g** Volcano plot showing the DEGs between amnion (AM-1 and AM-2) of the wild-type and mutant embryos. Gray areas indicate the expected group mean difference in random cell subsets (99.9th percentile) and a false discovery rate cutoff of 1%. Red labeling indicates that the gene is part of the ISL1 regulon identified by SCENIC. *P* values were calculated using a two-tailed Welch’s *t* test. The *x*-axis reports uncorrected *p* values. **h** Violin plot of the expression levels of *BMP4* and *WNT6* in the different cell types identified in (Fig. 1d) at Day 14 separated between wild-type (blue) and mutant (red) embryos. Epi epiblast, AM-1 amnion 1, AM-2 amnion 2, meso-1 mesoderm 1, meso-2 mesoderm 2, wt wild type, mt mutant, D dorsal, V ventral.

This was confirmed by immunofluorescent imaging of sectioned in vitro cultured mutant embryos at Day 14 (Fig. 4a–d), which showed no BRA (*TBXT*)-positive cells and only very few MIXL1-positive cells (Fig. 4b). Trophoblast staining positive for GATA3 and ExE-Mech staining positive for *VIM* showed similar distribution as compared to the wild type (Fig. 4a).

Differential gene expression analysis between the cells in the mesodermal clusters (meso-1 and meso-2) of mutant and wild-type embryos revealed 251 genes that were significantly downregulated in the mutants (Supplementary Data 5). This list was enriched for GO terms (biological processes) such as anterior/posterior pattern specification (GO:0009952), embryo development (GO:0009790), and mesoderm development (GO:0007498) (Fig. 4e). 163 of these genes were members of a large, highly interconnected STRING network (PPI enrichment *p* value: <1.0e – 16). Within this network, a STRING network cluster termed Wnt signaling pathway, and TGF-beta signaling pathway (CL:5698), showed the highest enrichment and was located in the center, suggesting that alterations in Wnt and/or TGF-beta signaling could be underlying the observed phenotype (Fig. 4f).

### Loss of ISL1 in amnion impairs signaling

The amnion has previously been suggested to serve as a signaling hub for gastrulation^38^ and, due to its high expression of *ISL1*, it is likely to be the primary affected tissue in the mutant embryos. Differential gene expression analysis in amniotic cells of mutant versus wild-type embryos revealed 184 significantly downregulated genes in the mutant amnion, of which a significant proportion were members of the identified ISL1 regulon (Fig. 4g and Supplementary Data 5). Among those was *BMP4*, a secreted member of the TGF-beta signaling pathway and a known downstream target of ISL1 in mice^39^, as well as *WNT6*, a secreted Wnt ligand (Fig. 4h). BMP4 was previously shown to be essential for murine mesoderm formation^40^ as well as for inducing primitive streak like cells from hESCs^35^, while WNT6 is known to be required later in embryonic development in somitogenesis in mouse and chick^41,42^.

Since the two amnion populations present in Day 14 embryos have distinct characteristics, differential gene expression analysis was also performed separately in AM-1 and AM-2 cells of mutant versus wild-type embryos. This analysis revealed that *BMP4* was among the most significantly downregulated genes in both populations (Supplementary Data 5). In addition, we noted that several genes belonging to the GO categories such as epithelium development (GO:0060429) and epithelial cell differentiation (GO:300855) were significantly downregulated in AM-2 cells of the mutant embryos (Supplementary Fig. 8). This suggests that the overrepresented AM-2 population in the mutant embryos consists largely of improperly formed amniotic epithelium. Indeed, immunofluorescent imaging of sectioned in vitro cultured mutant embryos at Day 14 for the amniotic epithelium marker GABRP showed no specific signal in the amnion region of the mutant embryos (Fig. 4d). In line with the findings from the genotyping, most cells in the amnion region stained negative for ISL1 (Fig. 4c).

Taken together, these findings suggest that loss of ISL1 causes altered signaling from the amnion, which results in failure to form mesoderm in the early NHP embryo eventually leading to embryonic lethality.

### *ISL1*-null hESC-derived amnion fails to induce mesoderm

**Fig. 5:**
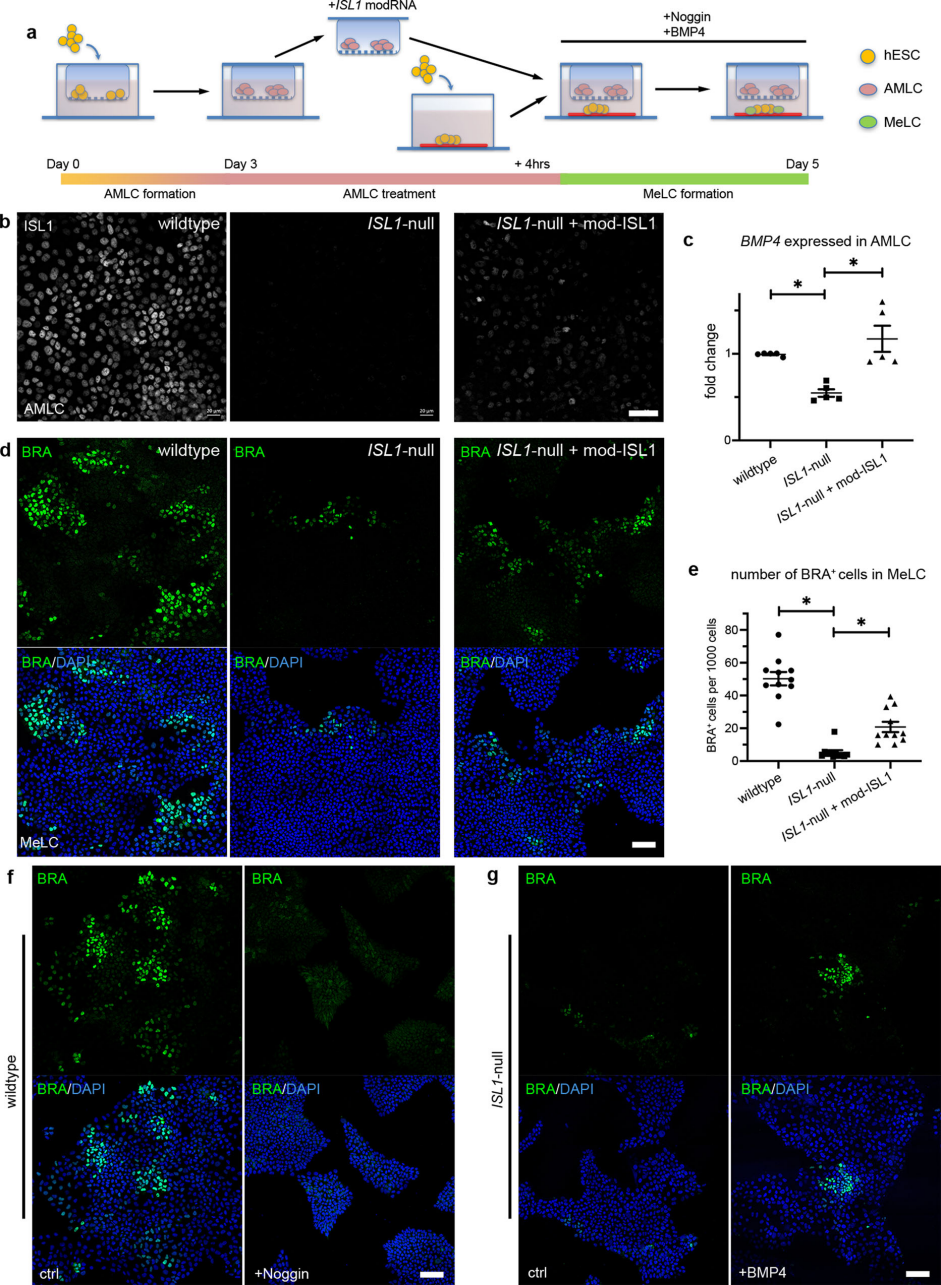
ISL1 in amnion-like cells regulates human mesodermal cell formation through BMP4. See also Supplementary Fig. 9-12. **a** Diagram of the transwell assay. **b** immunofluorescent staining for ISL1 in AMLCs. Scale bar 50 µm. *n* = 4 biologically independent experiments **c** expression of *BMP4* in AMLCs. Data are presented as mean ± SEM and analyzed by two-tailed Student’s *t* test. *n* = 6 biologically independent samples. *p value <0.05 (<0.0001 for wild type versus *ISL1*-null and 0.0002 for *ISL1*-null versus *ISL1*-null + mod-ISL1). **d** immunofluorescent staining for the mesoderm marker brachyury (BRA, green) in MeLCs. Nuclei stained with DAPI (blue). Scale bar 100 µm. **e** Quantification of BRA+ cells from (**d**). Data are presented as mean ± SEM and analyzed by two-tailed Student’s *t* test. *n* = 11 biologically independent samples. *p value <0.05 (<0.0001 for wild type versus *ISL1*-null and 0.0002 for *ISL1*-null versus *ISL1*-null + mod-ISL1). **f** immunofluorescent staining for BRA in wild-type MeLCs without or with Noggin treatment. Scale bar 100 µm. *n* = 3 biologically independent experiments. **g** Immunofluorescent staining for BRA in *ISL1*-null MeLCs without or with BMP4 treatment. Scale bar 100 µm. *n* = 6 biologically independent experiments. AMLCs amniotic ectoderm-like cells, MeLCs mesoderm-like cells.

To validate this conclusion and to investigate whether these findings also apply to humans, *ISL1-*null hESCs (*ISL1*-null) harboring the most abundant long deletion in the *ISL1* locus found in the mutant embryos (Supplementary Figs. 1a and 9a) were generated and analyzed in vitro (Fig. 5a). Amniotic ectoderm-like cells (AMLCs) derived from the *ISL1*-null showed a 50% reduction in *ISL1* messenger RNA (mRNA) (Supplementary Fig. 9b) and absence of ISL1 protein, which was abundantly expressed in wild-type hESC-derived AMLCs (Fig. 5b and Supplementary Fig. 9c). We noticed a slight, nonsignificant reduction in *WNT6* (Supplementary Fig. 9d) and a significant reduction in *BMP4* expression of ~50% (Fig. 5c), confirming the functional defect in *ISL1*-null derived AMLCs. In line with the findings from the in vitro cultured embryos, AMLCs derived from the *ISL1*-null failed to induce mesoderm-like cells (MeLCs) from hESCs shown by a strong reduction in the number of BRA (*TBXT*) expressing cells in the lower compartment (Fig. 5d, e). Notably, using a directed differentiation protocol towards MeLCs^35^, *TBXT* expression levels were similar between *ISL1*-null and wild type (Supplementary Fig. 10), highlighting that the failure to form mesoderm in the *ISL1*-null is a non-cell-autonomous defect caused by altered signaling from AMLCs.

Modified mRNA-based re-expression of ISL1 in *ISL1*-null AMLCs restored *BMP4* expression, confirming that BMP4 is acting downstream of ISL1 (Fig. 5c). Moreover, it significantly increased the capacity of the *ISL1*-null AMLCs to induce BRA-positive cells (Fig. 5d, e).

We observed similar effects using a modified mRNA encoding ISL1 with the same three amino acid deletion found in a small subset of cells in the *ISL1* mutant NHP embryos (Supplementary Fig. 11), suggesting that these cells had a functional ISL1 protein. The fact that the observed phenotype was consistent across all *ISL1* mutant embryos shows that this low-degree mosaicism of functional ISL1 was insufficient to sustain normal embryonic development.

### BMP4 rescues mesoderm formation in vitro

To further investigate whether the formation of MeLCs is indeed dependent on BMP4 signaling from the AMLCs, we inhibited BMP4 downstream signaling by using Noggin. This resulted in a significant reduction in the number of BRA-positive cells in wild-type hESCs (Fig. 5f and Supplementary Fig. 12a), mimicking the *ISL1*-null phenotype. Conversely, external addition of BMP4 in the *ISL1*-null led to a partial rescue of the phenotype, shown by a significant increase in the number of BRA-expressing cells (Fig. 5g and Supplementary Fig. 12b), suggesting that BMP4 signaling is responsible in part for the observed phenotype.

**Fig. 6:**
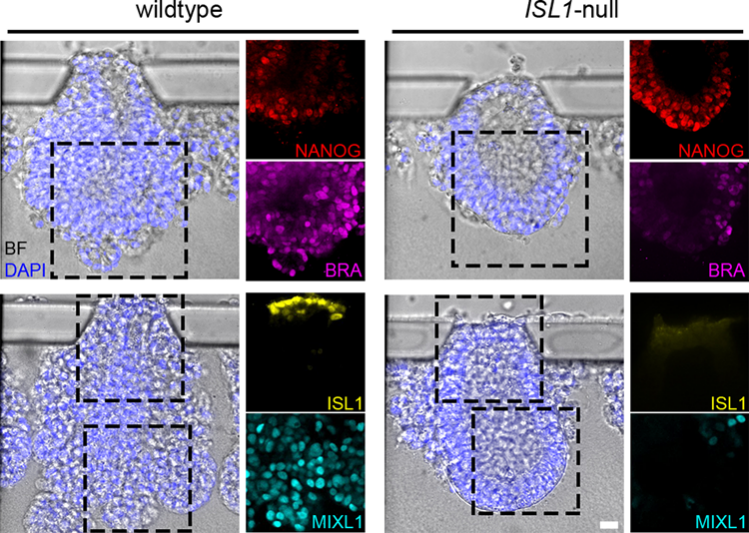
Embryonic-like sac assay. See also Supplementary Fig. 13. Brightfield (BF) images of embryonic-like sacs overlayed with nuclei stained with DAPI (blue) derived from wild-type (left side) and *ISL1*-null (right side) hESCs. Immunofluorescent staining for NANOG (red), BRA (magenta), ISL1 (yellow), and MIXL1 (cyan) are shown in the panels on the right-hand side of the corresponding brightfield images. *n* > 15 for each. Scale bar, 20 µm.

The capacity of *ISL1-*null and wild-type hESCs to self-organize into an embryonic-like sac was assessed in a microfluidic system that has been shown to faithfully recapitulate the peri-implantation development of the epiblast lineages^38^. We noticed that the high dose of BMP4 (50 ng/mL) used in the protocol of the original publication masked the phenotype in the *ISL1*-null, and thus we reduced the BMP4 concentration (25 ng/mL). With this reduced BMP4 dose, the wild-type cells still showed proper formation of embryonic-like sacs, adequate break of symmetry, and formation of MeLCs in the epiblast-like region as shown by positive staining for BRA and MIXL1 (Fig. 6 and Supplementary Fig. 13a, b). ISL1 showed specific staining in the AMLCs and was absent from other parts of the embryonic-like sac highlighting its potential as a robust marker for amnion in humans (Fig. 6 and Supplementary Fig. 13c). In the same region, we observed a signal for GABRP (Supplementary Fig. 13d). However, it did not show the membrane alignment present in the in vitro cultured embryos, which could suggest that the amnion in the embryonic-like sacs is not fully epithelialized yet.

In these conditions, *ISL1-*null cells were capable of self-organizing into embryonic-like sacs and broke symmetry similar to the wild type but failed to develop further. ISL1 signal was absent from the mutant AMLCs (Fig. 6 and Supplementary Fig. 13c). The epiblast-like cells mostly remained in a columnar shape with high levels of the pluripotency factor NANOG^24^ and stained negative for BRA and MIXL1 (Fig. 6 and Supplementary Fig. 13a, b), indicating failure to form MeLCs similar to the findings observed in the mutant embryos during in vitro culture. A similar phenotype was observed in embryonic-like sacs from wild-type hESCs when BMP4 downstream signaling was inhibited by using high-dose Noggin highlighting the importance of BMP4 signaling for MeLC formation (Supplementary Fig. 13e).

## Discussion

**Fig. 7:**
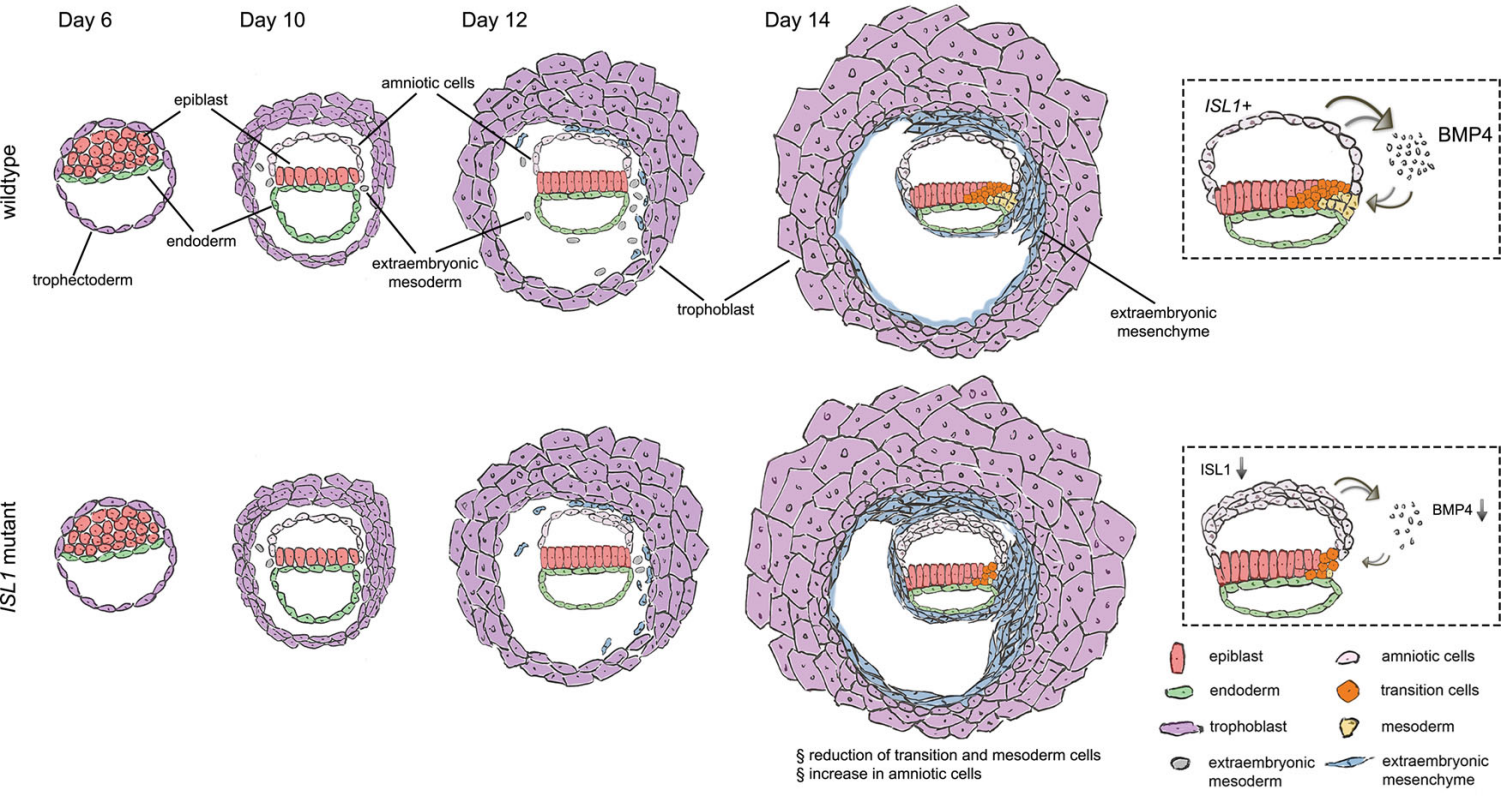
A summary scheme depicting the embryogenesis of wild-type and *ISL1* mutant embryos. In primate embryogenesis, the amnion forms a signaling center where *ISL1*-dependent BMP4 signaling drives streak and mesoderm formation.

In this study, we generated a high-resolution developmental roadmap of postimplantation NHP embryos and identified the amnion as a key signaling structure essential for mesoderm formation in primates. The transcription factor *ISL1* is highly expressed in primate amnion. Embryos with loss of function of ISL1 in most of the cells in the amnion fail to form mesoderm due to a reduction in BMP4 signaling and are not capable of giving rise to viable offspring (Fig. 7).

Notably, the role of ISL1 acting upstream of BMP4 seems to be a conserved pathway. The loss of *Isl1* in mice leads to normal gastrulation since it is not expressed in the mouse embryo before E7.0^7,33^. However, it is embryonically lethal at approximately E10.5 due to severe cardiac defects accompanied by a strong reduction in *Bmp4*^7^, suggesting that BMP4 is acting downstream of ISL1 in cardiac development. Similar observations linking *Isl1* and *Bmp4* were also made in mice during genital development^43^ and embryonic limb formation^44^.

It is known that the initiation of mesoderm formation is dependent on BMP4 signaling, which is provided by the extraembryonic ectoderm in mice^11,45^. The findings from our study suggest that this role is taken over by the amnion during primate embryogenesis. Mouse and primate embryos have a similar appearance before the implantation stage, although the transcriptome already differs in key aspects^10,46^. After implantation, the structural differences between mouse and primate embryos become more evident. Mouse embryos form a cup-like structure, while primate embryos acquire a disk-like shape and have a prominent amnion, which is absent in mouse embryos before gastrulation^28,47^. Although the signaling network guiding gastrulation appears to be largely conserved across species^37^, the findings from our study show that the anatomical differences in the early embryos are associated with the presence of alternative signaling centers.

Explanations for differences in gene essentiality between humans and mice span two ends of a spectrum. When an essential gene in mice fails to demonstrate a similar phenotype in humans, the disparate human phenotype could either be very subtle or the opposite, especially severe. We initially hypothesized that the low frequency of damaging *ISL1* variants reported in human cohorts was due to an important role in early embryonic development. Indeed, we detected its requirement during gastrulation. It is possible that comparable effects could also be observed for other genes, with a similar discrepancy between mouse and human phenotypes. This study highlights that in vitro cultured primate embryos are a powerful tool to model key steps of early human development and could make an important contribution in addressing these questions.

Further advances in the in vitro culture systems might enable us to support the embryo longer and to study early organogenesis, including the emergence of cardiac progenitor cells in lateral plate mesoderm. This would enrich our knowledge on human embryogenesis, help to identify causes for pregnancy loss and congenital malformations, and, eventually, open the avenue for new therapies.

## Methods

### Cynomolgus macaque

Healthy cynomolgus monkeys (*Macaca fascicularis*), ranging from 5 to 12 years old, were used in this study. All animals were housed either at the facility of Yunnan Key Laboratory of Primate Biomedical Research in China or at Astrid Fagræus laboratory in Karolinska Institutet in Sweden. Both facilities are accredited by AAALAC International. The ethics and all experimental protocols were approved in advance by the Institutional Animal Care and Use Committee of LPBR in China (KBI K001115033/01,01) and by the Jordbruksverket in Sweden (ethical permit number N277/14). Animals involved in this study were never used for other treatments.

### In vitro fertilization

NHP embryos were collected as described previously^48^. In brief, healthy female monkeys aged 5–8 years with regular menstrual cycles were selected as oocyte donors. Before superovulation, female animals were treated with recombinant human follicle-stimulating hormone for 8 days, and then were administered recombinant human chorionic gonadotropin alfa (rhCG) on day 9. Oocytes were collected by laparoscopic follicular aspiration 32–35 h after rhCG administration. MII (first polar body present) oocytes were performed with intracytoplasmic sperm injection to generate zygotes and the fertilization was confirmed by the presence of two pronuclei. Zygotes were cultured in embryo culture medium-9 (HECM-9) containing 10% fetal calf serum in a 37 °C incubator supplying 5% CO_2_ until the blastocyst stage. Blastocysts were then used for embryo transfer or postimplantation in vitro culture. The homemade HECM-9 contains polyvinyl alcohol (0.1 mg/mL), calcium chloride (1.9 mM), magnesium chloride (0.46 mM), potassium chloride (3.0 mM), sodium chloride (113.8 mM), sodium bicarbonate (25.0 mM), sodium lactate (4.5 mM), Minimum Essential Medium (MEM) amino acid, MEM nonessential amino acid, and gentamicin (10 mg/mL). Details of chemicals were listed in Supplementary Data 6.

### Embryo transfer and pregnancy diagnosis

Embryos were transferred into the oviducts of the matched recipient monkey as described in the previous study^49^. A total of 27 female monkey recipients with proper hormone levels of β-estradiol and progesterone were used as surrogate recipients. Each recipient received two to four blastocysts. The pregnancy was primarily diagnosed by ultrasonography at 2–3 weeks after embryo transfer. Clinical pregnancy and the number of fetuses were confirmed by fetal cardiac activity and the presence of gestation sacs. When terminating a pregnancy, a cesarean section was performed. Tissue from the umbilical cord, ear, and tail was collected for genotyping.

### Generation of *ISL1* NHP mutant embryos

NHP MII zygotes were injected with a mix of Cas9 protein and gRNAs. Intracytoplasmic injections were performed with a Nikon microinjection system under standard conditions. The embryos were cultured in HECM-9 supplemented with 10% fetal calf serum in a 37 °C incubator supplying 5% CO_2_. Genetically modified embryos with high quality from morula to blastocyst stage were used for further studies.

### In vitro embryo culture

To culture blastocyst beyond the implantation stage, we applied an optimized protocol based on the human embryo culture protocol from Zernicka-Goetz’s group^50^. Frozen NHP blastocysts were thawed right before culturing by using the Thawing Media (Kizatato) and cultured in blastocyst culture medium (Origio) for at least 4 h to recover. Blastocysts were then treated with acidic Tyrode’s solution to remove the zona pellucida and transferred to an ibiTreat 8-well μ-plate (Ibidi) containing 300 µL of pre-equilibrated in vitro culture medium 1 (IVC1). On the second day, 150 µL of IVC1 was carefully removed and 200 µL pre-equilibrated in vitro culture medium 2 was added. Blastocyst growth was monitored and the medium was changed every 2 days until the termination of experiments.

### Single-cell dissociation and RNA sequencing

NHP embryos at Days 10, 12, and 14 were washed with phosphate-buffered saline (PBS) and treated with TrypLE Express Enzyme for 30 min at 37 °C. After incubation, samples were gently dissociated into single cells by mouth pipetting.

Single cells were transferred into an RNase-free, low-cell-adhesion 1.5 mL tube and centrifuged at 300 × *g* for 5 min. The supernatant, containing some remaining cells, was transferred into a new tube for genotyping. The cell pellet was resuspended with 40 µL of PBS containing 2% bovine serum albumin. Cells were loaded into the 10x Genomics Chromium system within 30 min after dissociation. 10x Genomics v3 libraries were prepared according to the manufacturer’s instructions. Libraries were sequenced with a minimum coverage of 30,000 raw reads per cell on an Illumina NovaSeq with paired-end sequencing.

### Reads mapping, gene expression counting, and correction

Sequencing data were aligned and quantified by using the Cell Ranger Pipeline v3.1.0 against the ensemble genome build Macaca_fascicularis_5.0 release 96^51^. Ambient RNA contamination was estimated through the levels of choriogonadotropin expression in epiblast (*POU5F1*-positive) cells and removed from the count matrix using SoupX^52^. A gene was retained for analysis if it showed expression in at least three cells. Each sample was filtered based on the expression level of mitochondrial genes (<7.5%) and the number of expressed genes. Details on the estimated contamination in each sample, the filtering criteria, and the number of cells retained for the analysis are provided in Supplementary Fig. 2a.

### Reads mapping and gene expression counting of in vivo dataset

The raw, archived scRNA-sequencing data from in vivo cynomolgus embryos^19^ was downloaded from the GEO database under accession code GSE74767 and processed using TrimGalore v0.6.1. The reads passing quality control were aligned against the ensemble genome build Macaca_fascicularis_5.0 release 96 using STAR v2.5.3 and counted using featureCounts v1.5.2. Cells expressing at least 1000 genes were kept for integration with our dataset.

### Data integration, dimensionality reduction, and clustering

For analysis of the scRNA-sequencing data from the wild-type in vitro cultured embryos, we integrated the filtered, corrected count matrices of the different batches for each day separately using the reciprocal principal component analysis (PCA) approach implemented in the Seurat package v3.1.3^53,54^ based on 30 dimensions and 5000 anchor features. After integration, we performed PCA analysis on the integrated data followed by embedding it into low-dimensional space with UMAP as implemented in the R-package “uwot.” For clustering, the shared nearest-neighbor graph was constructed on the UMAP embedding by calling the FindNeighbors() function followed by the identification of clusters using the FindClusters() function, both part of the Seurat package. In some samples, this clustering approach separated large-cell groups into small subclusters with no distinct biological meaning in the context of this manuscript. In these cases, the clusters were recombined manually and both the unsupervised and the manually adjusted clustering was reported in the manuscript.

To integrate the scRNA-sequencing data from in vivo cynomolgus embryos^19^ with our dataset, we combined the three time points (Days 10, 12, and 14) from our dataset for each batch separately and did the same for the in vivo data from E08, E09, E13, and E14, resulting in three separate datasets. Normalization, scaling, and PCA was performed separately on each of these datasets, after which they were combined using the reciprocal PCA approach described above based on 30 dimensions and 2000 anchor features.

For the analysis of the wild-type and *ISL1* mutant embryos, the different batches were integrated separately for each day by the same reciprocal PCA approach outlined above based on 30 dimensions and 5000 anchor features using the wild-type datasets as reference. Dimensionality reduction and clustering were performed as described above.

To integrate our dataset with the scRNA-sequencing data from the in vivo human Carnegie stage 7 embryo^37^, *M. fascicularis* gene IDs were converted to *Homo sapiens* gene symbols according to the ortholog list from Ensemble. Normalization, scaling, and PCA was performed separately on each of the batches (wild-type and mutant embryos, Day 14) from our dataset and the human dataset separately, after which they were combined using the reciprocal PCA approach described above based on 30 dimensions and 2000 anchor features. Dimensionality reduction and clustering were performed as described above.

### Differential gene expression analysis

Mainly due to the differences in cell numbers, we observed a significant variation in sequencing depth between samples in our dataset (Supplementary Fig. 2). It has recently been shown that the effect of differences in the read depth on differential gene expression analysis can be minimized by using regularized negative binomial regression as implemented in the R-package SCtransform^55^. Thus, all differential gene expression analysis was performed using a *t* test on Pearson residuals after SCtransformation of the raw, filtered counts of the integrated Seurat object as implemented previously^55^. Gene expression data depicted throughout the manuscript in feature plots or violin plots are SCtransformed data. Expression data depicted in heatmaps ﻿are scaled, log-transformed expression values normalized to the total counts for each cell calculated through running the NormalizeData() function, followed by the ScaleData() function from the Seurat package.

For analysis of PPIs and enriched GO terms among differentially expressed genes, *M. fascicularis* gene IDs were converted to *H. sapiens* gene symbols according to the orthologue list from Ensemble. Interaction networks of differentially expressed genes were created using STRING v11.0 and analyzed for enriched GO terms as well as enriched STRING network clusters using standard settings^56^.

### Visualization of gene signatures

Scoring and visualization of gene signatures were performed using the Single Cell Signature Explorer v3.1^57^. Gene signatures were created by identifying orthologs for the genes that have been previously described to mark the naive and primed state of pluripotency in hESCs^21^ in the *M. fascicularis* genome according to the ortholog list provided from Ensemble through BioMart^58^.

### Pseudotime analysis

Pseudotime analysis was performed using Monocle3 v0.2^59–61^. ﻿The principal graph was learned on the UMAP embedding extracted from the integrated Seurat object. Differentially expressed genes were calculated on the raw, filtered count matrix extracted from the integrated Seurat object using the ﻿Moran’s I test implemented in the graph_test() function from the Monocle3 package. The genes were ranked according to their Moran’s I and the top 100 genes were selected for display in the heatmap and supplied as Supplementary Data 2.

### GRN analysis

GRN analysis was performed using the R-package SCENIC v1.1.2-2^32^ and the command line interface of the Python implementation pySCENIC. *M. fascicularis* gene IDs were converted to *H. sapiens* gene symbols according to the ortholog list from Ensemble. The raw, filtered count matrix extracted from the integrated Seurat object was prefiltered and genes with at least 39 counts, equal to at least three UMI counts in 1% of the cells, present in at least 13 cells, equal to 1% of the cells, were used as input for the CLI. The human motif collection v9 and the cisTarget databases for hg38 were used in the pipeline and downloaded from https://resources.aertslab.org/cistarget/. Thresholds used for binarization were calculated depending on the distribution of the AUC values, which were tested with Hartigan’s Dip Test. For unimodal distributions, the threshold was set as mean plus two standard deviations. For bimodal distributions, the threshold was defined as the trough in between two peaks, which was solved as a minimization problem on the kernel smoothed density^62^. After binarization, regulons showing activity in at least 1% of the cells were included in the downstream analysis.

### Culture of hESCs

hESCs used in this study include HES-3 human ES cells and H9 human ES cells (WiCell). Genetic modification of the *ISL1* locus to generate *ISL1-*null hESCs was performed on HES-3 cells by applying CRISPR/Cas9 with the same gRNAs used in NHP blastocysts. Two *ISL1*-knockout cell lines were generated, named *ISL1*-null_c15 and *ISL1-*null_c51, and genotyped (Supplementary Fig. 1a). All cell lines were validated as karyotypically normal by Cell Guidance Systems (UK) (Supplementary Fig. 9a). Mycoplasma contamination test was performed regularly as negative. hESCs were maintained in a standard feeder-free culture system using mTeSR1 medium on 1% Matrigel or Essential 8 medium on 1% vitronectin. Cells were passaged every 4–5 days and visually examined during each passage to ensure the absence of spontaneous differentiation. Work with hESCs was carried out according to Swedish legislation following the recommendations of the Swedish National Council on Medical Ethics.

### Genotyping

For bulk genotyping, cells leftover from the single-cell sequencing were pooled. Genomic DNA was extracted by the phenol–chloroform method. DNA fragments covering both gRNA target sites were PCR amplified and ligated to TOPO TA cloning vectors. At least 50 bacteria clones per sample were picked for Sanger sequencing and used to estimate the genomic mutation rate.

For single-cell genotyping, dissociation was performed as described further above. Cells were manually picked and transferred into 5 µL of QuickExtract solution to extract DNA, following the manufacturer’s protocol. After the first round of high-fidelity PCR amplification (Phusion, NEB) using primers annealing to the *ISL1* region that covers the two gRNA binding sites, PCR products were purified using AMPure XP magnetic beads. Later, the second round of PCR amplification was performed using the same primers. The PCR products were analyzed by DNA agarose gel electrophoresis. Primers used for genotyping are listed in Supplementary Data 6.

### Off-target assay

Cas-OFFinder was applied to search for potential off-target sites with maximal two mismatches and two bulges^18^. Among all off-target candidates of both gRNAs, targets located on gene exons were selected for testing. The DNA fragments of target sites were PCR amplified and the sequences were confirmed by Sanger sequencing. Primers are listed in Supplementary Data 6.

### RNA extraction and quantitative real-time PCR

Total message RNA was extracted by Direct-zol RNA MiniPrep Kits and cDNA libraries were prepared using GoScript Reverse Transcriptase. Quantitative real-time PCR was performed by PowerUp SYBR Green Master Mix on ABI 7500Fast machine. Primers and chemicals are listed in Supplementary Data 6.

### Transwell assay

The transwell assay was performed based on previous work by Zheng et al.^38^. In brief, it was performed on Transwell 12-well plates with permeable polyester membrane inserts (0.4 μm, Corning). The membrane inserts were coated with 1% Geltrex diluted in Dulbecco’s Modified Eagle’s Medium/Nutrient Mixture-12 for 1 h before use. hESCs were collected and resuspended in the culture medium containing Y-27632 (10 μM) and seeded onto the membrane insert at a density of 3 × 10^4^ cells/cm^2^. Eighteen hours after seeding, the culture medium was changed to E6 medium supplemented with basic fibroblast growth factor (bFGF) (20 ng/mL) and BMP4 (50 ng/mL) and cultured for 48 h. On day 3, undifferentiated hESCs were collected, resuspended in E6 supplemented with bFGF (20 ng/mL), and seeded at a density of 9 × 10^4^ per well on freshly coated 12-well plates. The membrane inserts were washed with E6 + bFGF and transferred on top of the reseeded hESCs. Cells were collected after 48 h of analysis. Two wild-type hESC lines (HES-3 and H9) and two *ISL1*-null lines were used in this assay. Both of the wild-type cell lines showed comparable results, as did the two *ISL1-*null lines.

Noggin inhibition, BMP4 rescue, and *ISL1* modified mRNA rescue were carried out in the transwell assay as follows. Noggin (500 ng/mL) and BMP4 (20 ng/mL) were administered into E6 + bFGF in the lower part of the transwell inserts from day 3, after transferring inserts on top of hESCs. The *ISL1* modRNA carrying a GFP reporter sequence was designed to encode either a wild-type ISL1 (mod-ISL1wt) or an ISL1 with a three amino acid deletion (mod-ISL1∆9nt) and in vitro synthesized according to the previous work^63^. Briefly, modRNA was synthesized in vitro by using T7 RNA polymerase-mediated transcription from linearized DNA templates incorporating generic 5′-untranslated region (UTR), targeted gene, 3′-UTR, and a poly-A tail. The modRNA was then purified using Amion MEGAclear spin columns and treated with Antarctic phosphatase for 30 min at 37 °C. After repurification, the modRNA was quantified by Nanodrop and diluted to ~1 μg/μl for use. One milligram of purified modRNA was introduced into each sample of AMLCs on insert membrane on Day 3 by Lipofectamine RNAiMAX and incubated for 4 h. The inserts were then transferred on top of hESCs to induce MeLC formation.

### Directed differentiation of hESCs to MeLCs

Differentiation of hESCs to MeLCs was done in chemically defined media as previously described^35^. In brief, the posterior primitive streak was induced by the addition of bFGF (20 ng/mL), the phosphoinositide 3-kinase inhibitors LY294002 (10 μM) and BMP4 (10 ng/mL). The anterior primitive streak was induced with the same factors, and, additionally, activin A (50 ng/mL). After 40 h, cells were harvested. RNA extraction, reverse transcription, and quantitative real-time PCR were performed as detailed further above with 200 ng RNA as input for RT reaction. Primers and chemicals are listed in Supplementary Data 6.

### Microfluidic assay of embryonic-like sac

This assay was performed as previously described^64^. Briefly, the microfluidic device is fabricated by bonding a polydimethylsiloxane structure layer to a coverslip. Geltrex is diluted to 70% using E6 medium and loaded into the central gel channel separated from the side channels by trapezoid-shaped supporting posts. Upon gelation, the Geltrex matrix would generate concave Geltrex pockets between supporting posts for cell seeding. hESCs suspended in mTeSR1 medium was introduced into the cell loading channel and allowed to settle and cluster in the gel pockets. After hESC cluster formation, mTeSR1 medium was replaced by a basal medium (E6 and 20 ng/mL bFGF) and 20 ng/mL BMP4 was supplemented only into the cell seeding channel. After 18 h of BMP4 stimulation, the BMP4 medium was replaced by the basal medium. The microfluidic devices were fixed 48 h after the hESCs clusters were exposed to BMP4. To test the BMP4 signaling function, Noggin (50 and 500 ng/mL) was supplemented into the basal medium into the cell loading channel for 48 h. The hESC clusters were then fixed and immunofluorescent staining was performed.

### Cryosection of NHP embryos

Day 14 NHP embryos were fixed by 2% paraformaldehyde overnight at 4 °C and then washed by PBS. Dehydrate the fixed embryos with 30% sucrose overnight at 4 °C, and then embedded in OCT and froze in liquid nitrogen. Frozen blocks were performed cryosection on Cryostat (Thermo CryoStar NX70) according to the manufacturer’s protocol. Two wild-type and three *ISL1* mutant embryos were used for cryosection and then performed immunofluorescent staining.

### Immunohistochemistry

Immunohistochemistry of cells from the transwell assay was performed following standard procedures. Briefly, cells were fixed in 2% paraformaldehyde for 30 min at room temperature and washed with PBS. Cells were blocked in blocking buffer (serum diluted in PBS with 0.1% Triton X-100) for 1 h and then incubated with primary antibodies diluted in blocking buffer overnight at 4 °C. Cells were washed with PBS supplemented with 0.1% Tween-20 (PBS-T) and incubated with secondary antibodies diluted in blocking buffer for 2 h at room temperature. The embryonic-like sac structure was assessed by immunohistochemistry using the same protocol as published before^64^. Briefly, cells were fixed in 4% paraformaldehyde (PFA) for 12 h and then permeabilized in 0.1% sodium dodecyl sulfate (SDS) for another 3 h. After blocking in 4% serum/PBS, samples were incubated with primary antibody solution for 24 h at 4 °C, followed by incubation with secondary antibody solution for another 24 h at 4 °C. After staining, all samples were washed with PBS-T and then mounted for imaging. The dilution for the primary antibodies used in this study were anti-ISL1 (1:300), anti-OCT4 (1:200), anti-NANOG (1:400), anti-BRA (1:600), anti-MIXL1 (1:100), anti-GABRP (1:300), anti-SOX17 (1:400), anti-GATA3 (1:900), anti-VIM (1:2000), and anti-TFAP2c (1:100). Secondary antibodies were used in a dilution of 1:500. Details of the antibodies are listed in Supplementary Data 6. Confocal micrographs were acquired by Zeiss 700 LSM confocal microscope or Olympus spinning-disc confocal microscope (DSUIX18) equipped with an EMCCD camera (iXon X3, Andor). The brightfield morphologic images of embryonic-like sacs were acquired by Zeiss Observer.Z1 microscope equipped with a monochrome CCD camera (AxioCam, Carl Zeiss MicroImaging). Images were analyzed by iMaris.

### Western blot

Western blot was performed according to standard protocol. Briefly, to extract the protein from AMLCs, the cells were washed twice with cold PBS and RIPA buffer supplemented with protease and Phophatase Inhibitor Cocktail was added. Lysates were centrifuged at 14000 × g for 15 min at 4 °C and the supernatants were transferred to new tubes. BCA assay was performed to quantify the concentration of protein. After dilution, samples were aliquoted, mixed with LDS sample buffer, and denatured. Ten milligrams of total protein was loaded to run a 10% Bis-Tris protein gel in MOPS SDS running buffer and then transferred to a 0.2 μm PVDF membrane. The membrane was blocked with 5% milk/TBS-T for 30 min at room temperature and incubated with diluted anti-ISL1 (1:1000) or anti-β-actin (1:5000) antibody in blocking buffer overnight at 4 °C. The membrane was washed with TBS-T 10 min three times and then incubated in diluted horseradish peroxidase-conjugated secondary antibody (1:10,000) for 1 h at room temperature. After washing with TBS-T, the membrane was treated with ECL substrate and imaged using ChemiDoc Imaging Systems (Bio-Rad). Antibodies and chemicals used are listed in Supplementary Data 6. The full scan blot is presented in the Source data file.

### Quantification and statistical analysis

Values are shown as the mean value ± SEM. Continuous data were analyzed using Student’s *t* test. If not stated differently, *p* values or adjusted *p* values (where appropriate) <0.05 were considered statistically significant. Each experiment was repeated independently at least three times. Details on the samples (e.g., number of biological replicates) are indicated in figure legends. Graphs were generated using Prism or R.

### Reporting summary

Further information on research design is available in the Nature Research Reporting Summary linked to this article.

## Supplementary information

Supplementary Info

Supplementary Data 1

Supplementary Data 2

Supplementary Data 3

Supplementary Data 4

Supplementary Data 5

Supplementary Data 6

Reporting Summary

## Data Availability

The raw data, unfiltered count matrix, and processed count matrix generated in this study have been deposited in the Gene Expression Omnibus (GEO) database under accession code GSE148683. The single-cell RNA-sequencing data from the in vivo cynomolgus embryos^19^ used in this study are available in the GEO database under accession code GSE74767. The single-cell RNA-sequencing data from the in vivo human embryo^37^ used in this study have not been deposited by the authors in a publicly available database yet, but the processed data is available through a web resource [http://www.human-gastrula.net/]. Full-sized scans of western blots and immunofluorescent images are available in the source data file. Source data are provided with this paper.

## Source data

source data
