# Supplementary material for: Amnion signals are essential for mesoderm formation in primates
Source: Nat Commun. 2021 Aug 26;12:5126. doi: 10.1038/s41467-021-25186-2 (PMC8390679; doi:10.1038/s41467-021-25186-2)
Supplement: Supplementary file 1 — Supplementary Info [file 41467_2021_25186_MOESM1_ESM.pdf]

Supplementary Information

# Amnion signals are essential for mesoderm formation in primates

Ran Yang[#], Alexander Goedel[#], Yu Kang[#], Chenyang Si, Chu Chu, Yi Zheng, Zhenzhen Chen, Peter J. Gruber, Yao Xiao, Chikai Zhou, Nevin Witman, Elif Eroglu, Chuen-Yan Leung, Yongchang Chen, Jianping Fu, Weizhi Ji[*], Fredrik Lanner[*], Yuyu Niu[*], Kenneth R. Chien[*]

# Authors contributed equally.
* Correspondence: kenneth.chien@ki.se, fredrik.lanner@ki.se, niuyy@lpbr.cn, wji@lpbr.cn.

**This PDF file includes:**
Supplementary Figures 1-13
Supplementary Data 1-6

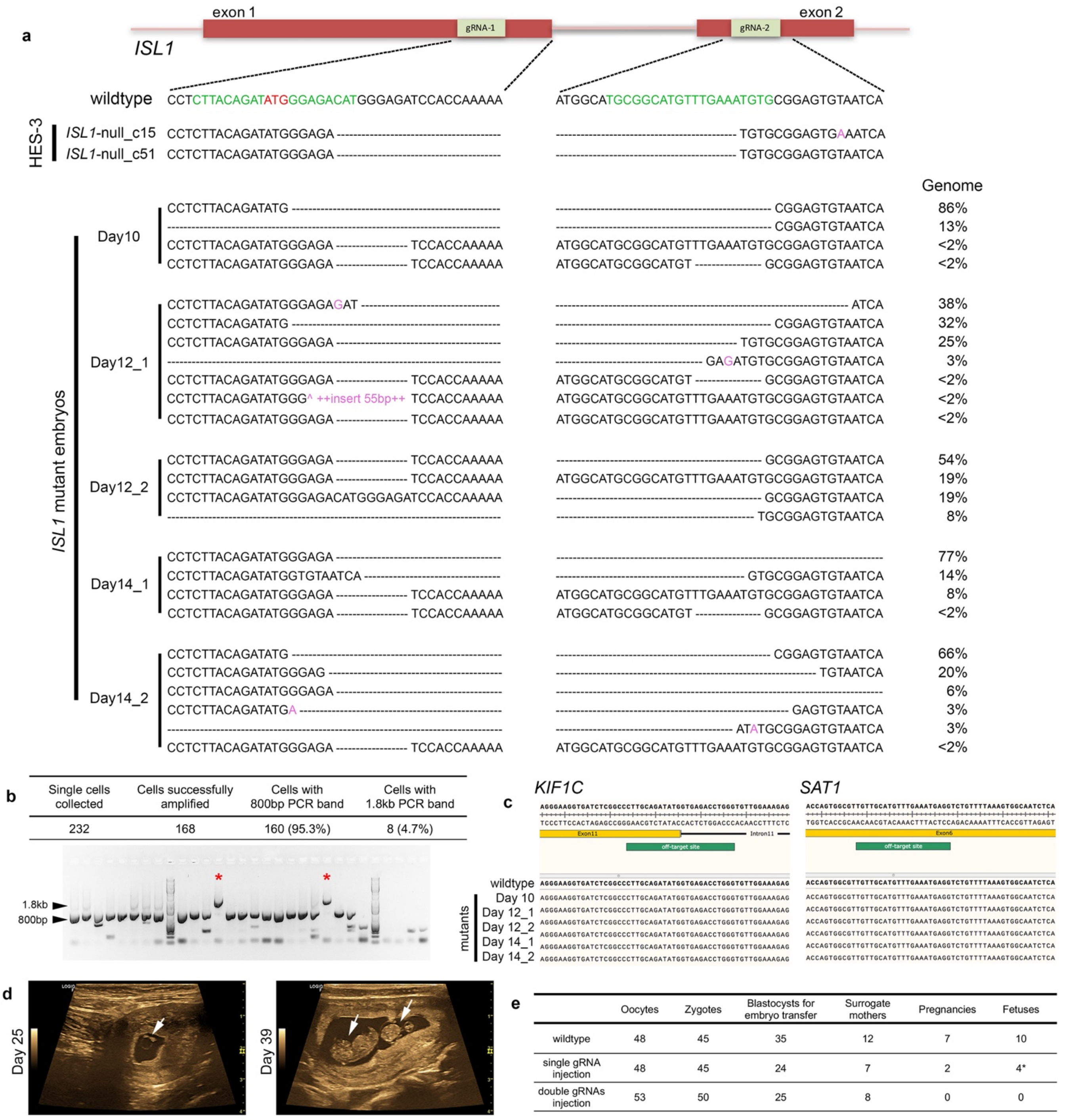


**Supplementary Fig. 1: Genotyping, off-target assay and embryo transfer of *ISL1* mutants.** Related to Fig. 1 and Fig. 5.

**a**, bulk genotyping of two *ISL1*-null hESC-lines and all *ISL1* mutant embryos used for scRNA sequencing. Green sequences mark the gRNA target regions, dotted lines show deletions and pink color indicates insertions. Start codon of *ISL1* is marked in red. Numbers on right side are percentages of each mutation type in each sample. **b,** PCR bands of single cell genotyping from an *ISL1* mutant embryo. The DNA gel is a representative image of the gels used to create the summary table above. Each lane of the DNA gel represents one single cell. Asterisks indicate cells with 1.8kb band. **c**, off-target analysis of *ISL1* mutant embryos. **d**, ultrasound imaging for pregnancy diagnosis. Days correspond to fetal age. Arrows indicate fetus. **e**, summary of embryo transfer. Asterisks indicate all four fetuses had an unmodified *ISL1* locus.

**a**

| type | day | batch | embryos per batch | cells from cellranger | estimated % contamination | minimal gene # used for filtering | cells after filtering |
|---|---|---|---|---|---|---|---|
| wt | D10 | B1 | 2 | 758 | 3.22 | 2500 | 658 |
| wt | D10 | B2 | 3 | 874 | 0.20 | 2500 | 670 |
| mt | D10 | B1 | 3 | 1063 | 1.04 | 2500 | 769 |
| wt | D12 | B1 | 1 | 510 | 2.27 | 2500 | 325 |
| wt | D12 | B2 | 2 | 1333 | 1.08 | 1500 | 981 |
| mt | D12 | B1 | 3 | 7263 | 4.35 | 1500 | 6018 |
| mt | D12 | B2 | 1 | 8284 | 7.99 | 1500 | 5842 |
| wt | D14 | B1 | 2 | 4685 | 2.12 | 1500 | 3176 |
| wt | D14 | B2 | 2 | 1731 | 1.02 | 1500 | 1384 |
| mt | D14 | B1 | 3 | 12951 | 6.90 | 1000 | 9625 |
| mt | D14 | B2 | 1 | 7719 | 3.22 | 1500 | 3882 |

**b**

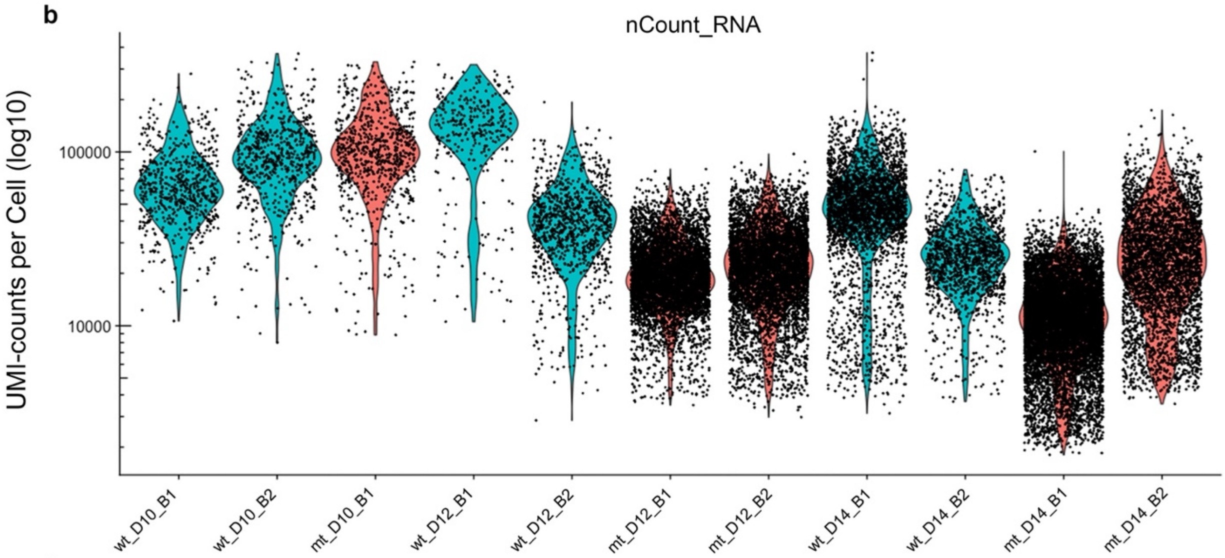


**c**

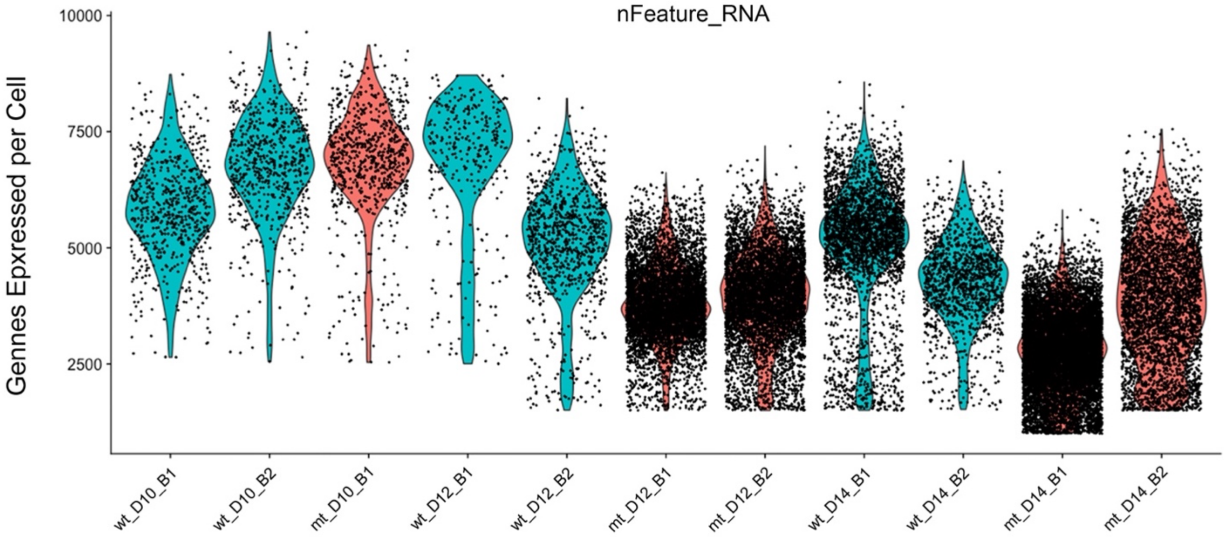


**Supplementary Fig. 2: Cell numbers, filtering and quality control of cells used in scRNA sequencing analysis.** Related to Fig. 1 and Fig. 3.

**a**, table containing information on cell numbers, filter criteria for each batch of *in vitro* cultured embryos used for scRNA sequencing. **b**, UMI counts per cell across the different batches. **c**, gene counts per cell across the different batches.

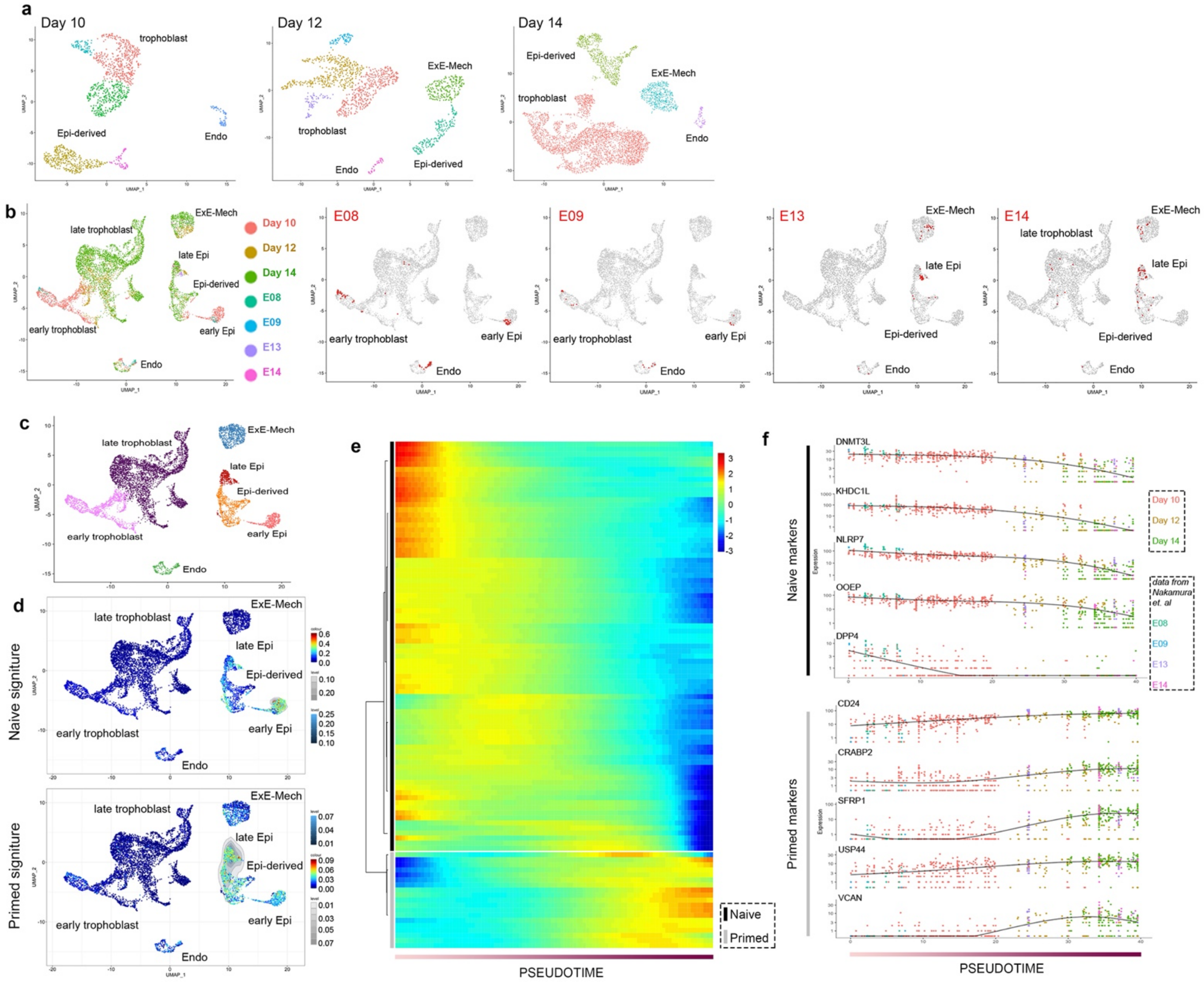


**Supplementary Fig. 3: Clustering, cell type identification and expression of marker genes in wildtype embryos.** Related to Fig. 1 and 2, and Supplementary Table 1.

**a**, unsupervised clustering results of all cells from wildtype embryos used for cell type identification as shown in (Fig. 1b). **b,** UMAP plot of the integrated dataset of all cells from the *in vitro* cultured embryos (Day 10, Day 12 and Day 14) with cells from the *in vivo* NHP embryos (E08, E09, E13 and E14) colored by datasets and days. Red dots mark cells from the *in vivo* NHP embryos on each day. **c**, UMAP plot of all cells from (**b**) colored by cell type. **d,** visualization of the single cell signature score reflecting the naïve and the primed state of pluripotency on the UMAP plot of the integrated dataset. **e**, heatmap showing the scaled expression of 100 DEGs across pseudotime in epiblast development selected by the Moran's I statistics (two-sided). Unsupervised clustering of genes into a black cluster containing genes downregulated over pseudotime and a grey cluster containing genes upregulated over pseudotime. **f**, gene expression of 5 exemplary genes downregulated over pseudotime (black cluster) and 5 exemplary genes upregulated over pseudotime (grey cluster) from (**e**). Black line showing the fitted expression trend for each gene over pseudotime. Epi, epiblast; Epi-derived, epiblast and epiblast derived cells; ExE-Mech, extraembryonic mesenchyme; Endo, endoderm.

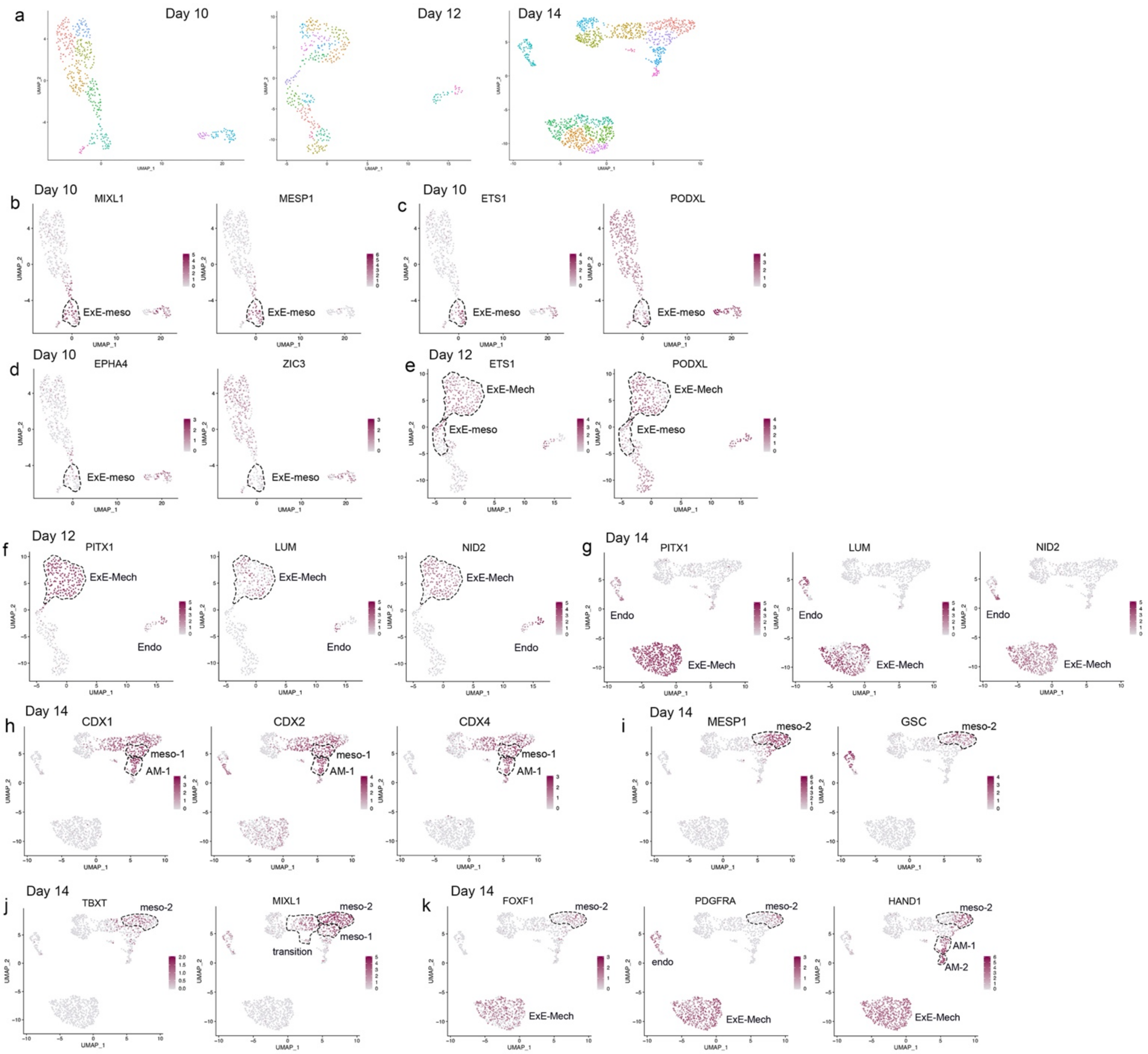


**Supplementary Fig. 4: Clustering, cell type identification and expression of marker genes from wildtype embryos.** Related to Fig. 1.

**a,** unsupervised clustering results of cells excluding trophoblast from wildtype embryos used for cell type identification as shown in (Fig. 1d). **b-d,** expression of marker genes for **(b)** mesoderm, **(c)** extraembryonic mesoderm and **(d)** embryonic mesoderm at Day 10 depicted on UMAP plots as shown in (Fig. 1d). **e,** expression of extraembryonic mesoderm markers at Day 12 depicted on UMAP plots as shown in (Fig. 1d). **f-g,** expression of genes shared between extraembryonic mesenchyme and endoderm at **(f)** Day 12 and **(g)** Day 14. **h,** expression of marker genes for mesoderm 1 at Day 14. **i-j,** expression of marker genes for mesoderm 2 at Day 14. **k,** expression of genes shared between mesoderm and extraembryonic mesenchyme at Day 14. Endo, endoderm; ExE-meso, extraembryonic mesoderm; ExE-Mech, extraembryonic mesenchyme; AM-1, amnion 1; AM-2, amnion 2; meso-1, mesoderm 1; meso-2, mesoderm 2.

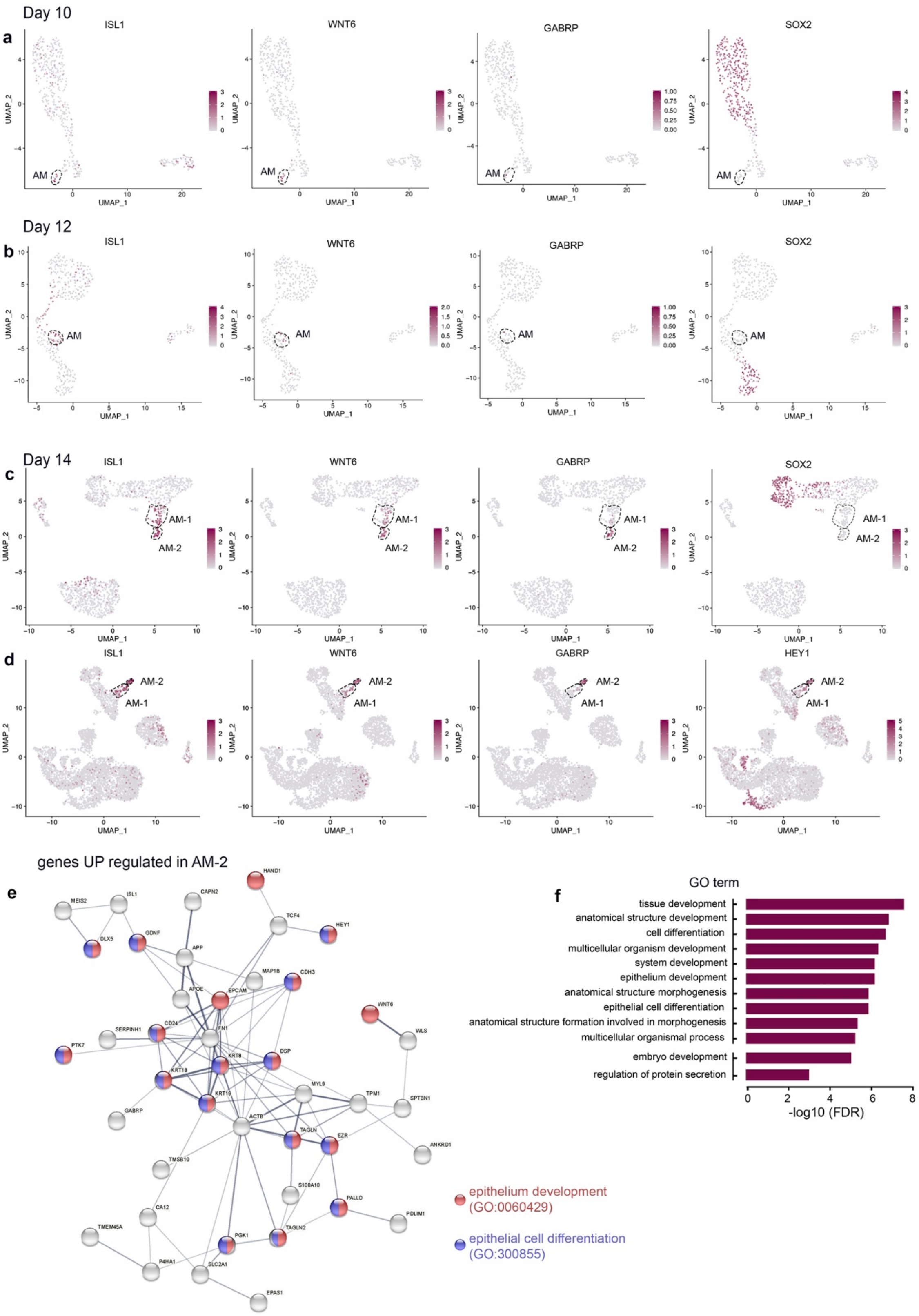

**Supplementary Fig. 5: Analysis of amnion populations.** Related to Fig. 1 and Supplementary Table 2.

**a-c,** gene expression in amnion cells depicted on the UMAP plots as shown in (Fig. 1d) at **(a)** Day 10, **(b)** Day 12, and **(c)** Day 14. **d**, expression of amnion marker genes at Day 14 depicted on the UMAP plot of the full dataset as shown in (Fig. 1b). **e,** STRING network of all genes significantly upregulated in cells of the AM-2 cluster compared to AM-1 in wildtype embryos at Day 14. Nodes not connected to the main network have been removed. Nodes belonging to the GO category epithelium development colored in red; nodes belonging to the GO category epithelial cell differentiation colored in blue. **f,** GO categories enriched among significantly upregulated genes in cells of the AM-2 cluster compared to AM-1 in the wildtype embryos at Day 14 ordered by false discovery rate (FDR). n = 4 wildtype and 4 mutant embryos collected in 2 batches. AM-1, amnion 1; AM-2, amnion 2.

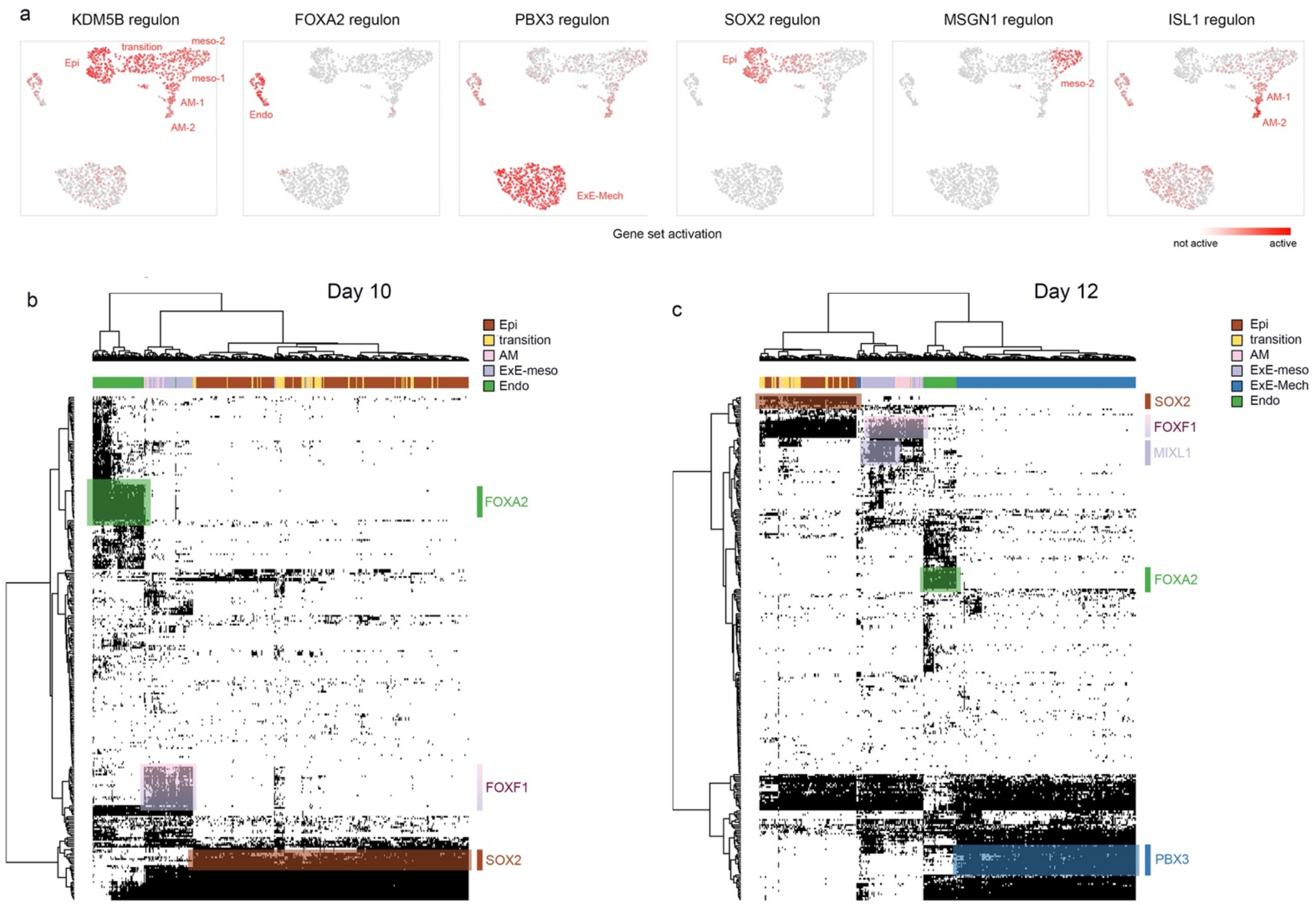


**Supplementary Fig. 6: SCENIC analysis.** Related to Fig. 2.

**a**, non-binarized gene set activity of the selected regulons at Day 14 in the different cell types depicted on the UMAP plot from (Fig. 1d). **b-c**, binary activity matrix of regulons identified at Day 10 and Day 12 by gene regulatory network inference active in at least 1% of the cells clustered unsupervised. **(b)** Day 10 and **(c)** Day 12. Epi, epiblast; Endo, endoderm; ExE-meso, extraembryonic mesoderm; ExE-Mech, extraembryonic mesenchyme; AM, amnion; AM-1, amnion 1; AM-2, amnion 2; meso-1, mesoderm 1; meso-2, mesoderm 2.

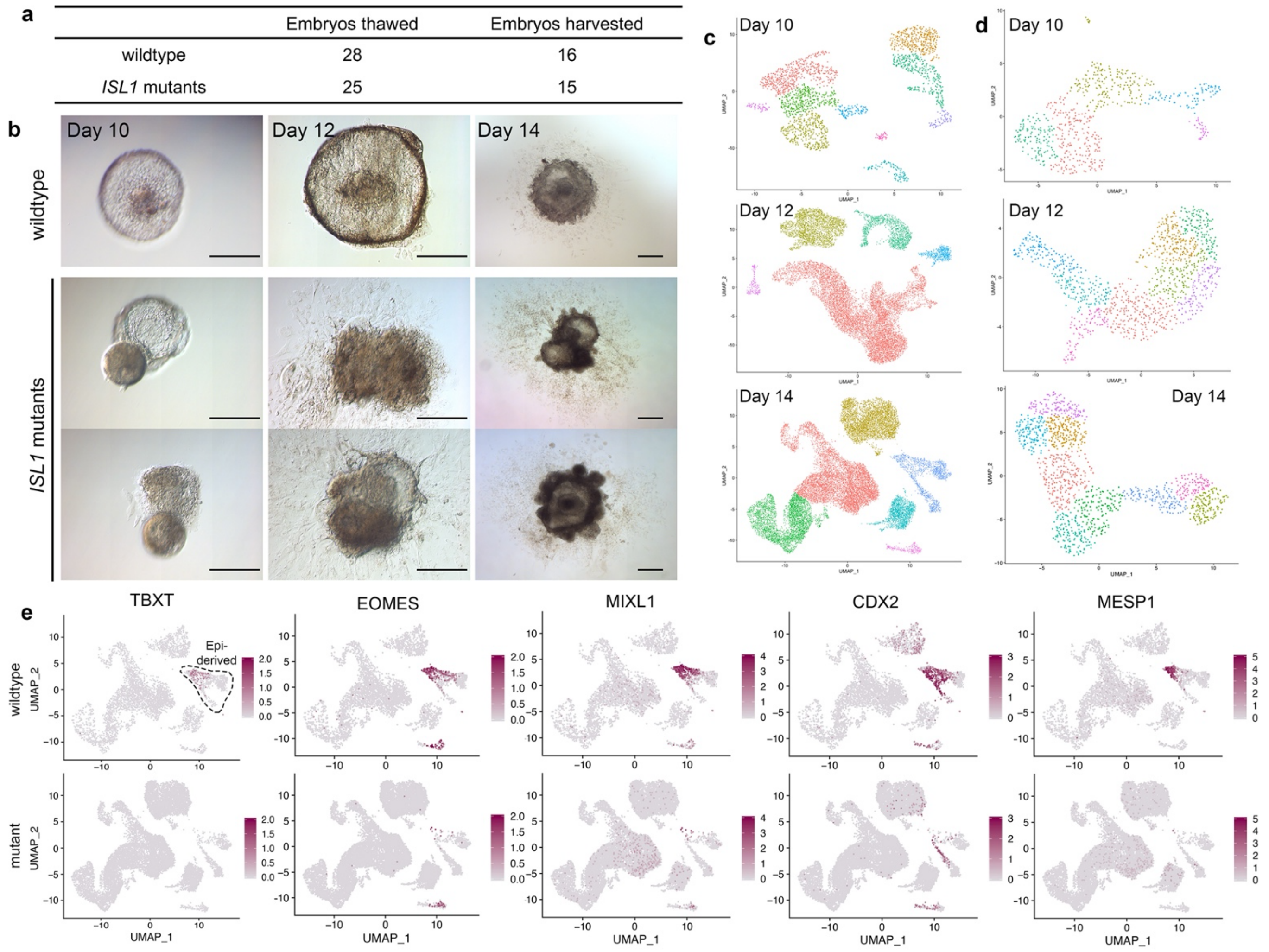


**Supplementary Fig. 7: Morphology and integrated analysis of wildtype and *ISL1* mutant embryos**. Related to Fig. 3.

**a**, summary of embryos used for *in vitro* culture. **b**, morphology of *in vitro* cultured NHP embryos. Scale bar, 50 µm, n = 15 wildtype and 14 mutant embryos. **c**, unsupervised clustering results of all cells from the integrated analysis of wildtype and mutant embryos used for cell type identification as shown in (Fig. 3b). **d**, unsupervised clustering results of cells mapping to the epiblast (including its derivatives) from the integrated analysis of wildtype and mutant embryos used for cell type identification as shown in (Fig. 3c). **e**, expression of mesoderm markers depicted on the UMAP plot of the full dataset from the integrated analysis of wildtype and mutant embryos at Day 14. Epi-derived, epiblast and epiblast derived cells.

genes DOWN regulated in AM-2 of *ISL1* mutant embryos

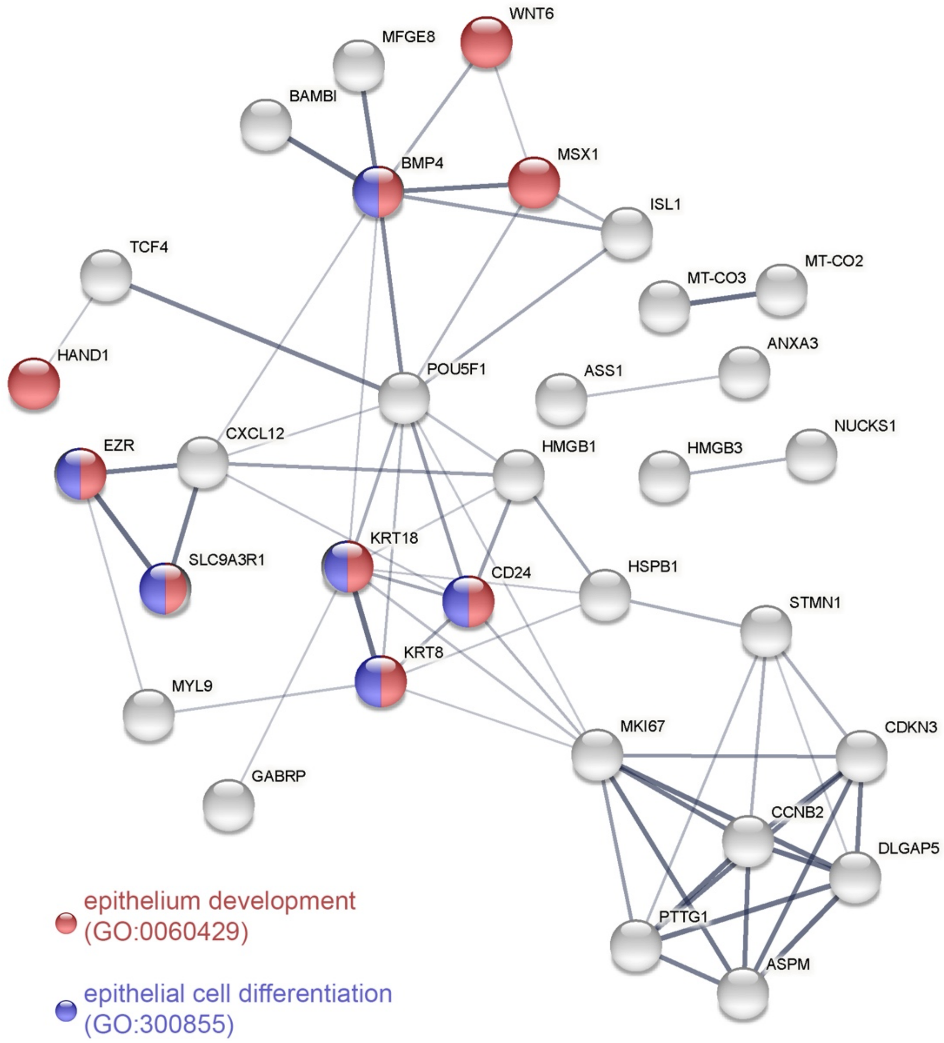


**Supplementary Fig. 8: STRING analysis of differentially expressed genes in AM-2 of wildtype and *ISL1* mutant embryos**. Related to Fig. 3.

STRING network of all genes significantly downregulated in cells of the AM-2 cluster in *ISL1* mutant embryos compared to the wildtype embryos at Day 14 (PPI enrichment p-value: 1.59e-12). Nodes not connected to the main network have been removed. Nodes belonging to the GO category epithelium development (GO:0060429) colored in red (FDR for enrichment = 0.0095); nodes belonging to the GO category epithelial cell differentiation (GO:300855) colored in blue (FDR for enrichment = 0.0156). AM-2, amnion 2.

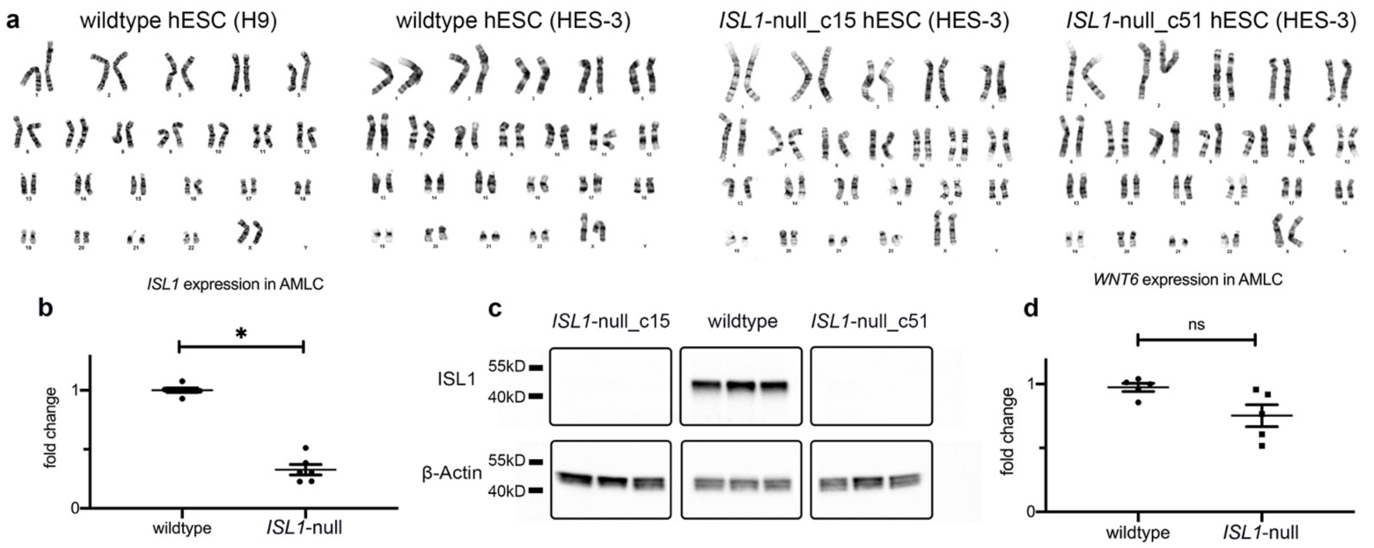


**Supplementary Fig. 9: transwell assay with wildtype and *ISL1*-null hESCs.** Related to Fig. 5.

**a**, karyotyping of the wildtype and the *ISL1*-null hESCs. **b**, expression of *ISL1* in wildtype and *ISL1*-null AMLCs. Data are presented as mean ± SEM and analyzed by two-tailed student's t-test. n = 6 biologically independent samples. * p-value < 0.0001. **c**, western blot for ISL1 and β-Actin (loading control) in wildtype and *ISL1*-null AMLCs. n = 4 biologically independent experiments. **d**, expression of *WNT6* in wildtype and *ISL1*-null AMLCs (p-value for difference = 0.0589). Data are presented as mean ± SEM and analyzed by two-tailed student's t-test. n = 5 biologically independent samples.

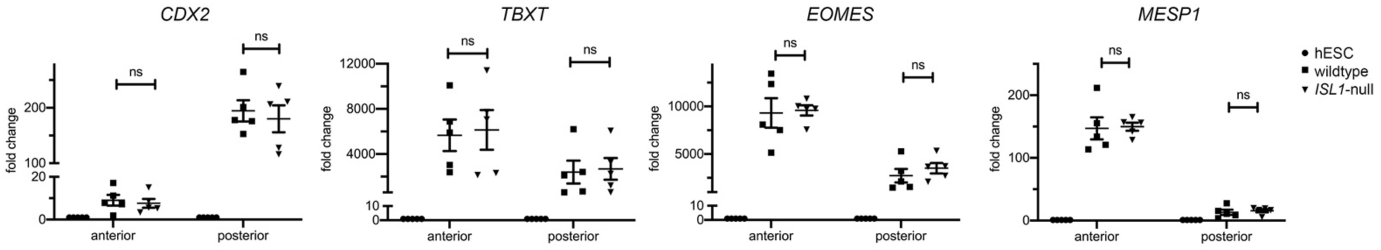


**Supplementary Fig. 10: *in vitro* assay for directed differentiation towards mesodermal cells.** Related to Fig. 5.

Expression of mesodermal marker genes (*CDX2, TBXT*, *EOMES*, *MESP1*) in mesoderm-like cells at 40h after induction towards anterior or posterior primitive streak from wildtype and *ISL1*-null hESCs. Data are presented as mean ± SEM and analyzed by two-tailed student's t-test. n = 5 biologically independent samples, all p-values > 0.05.

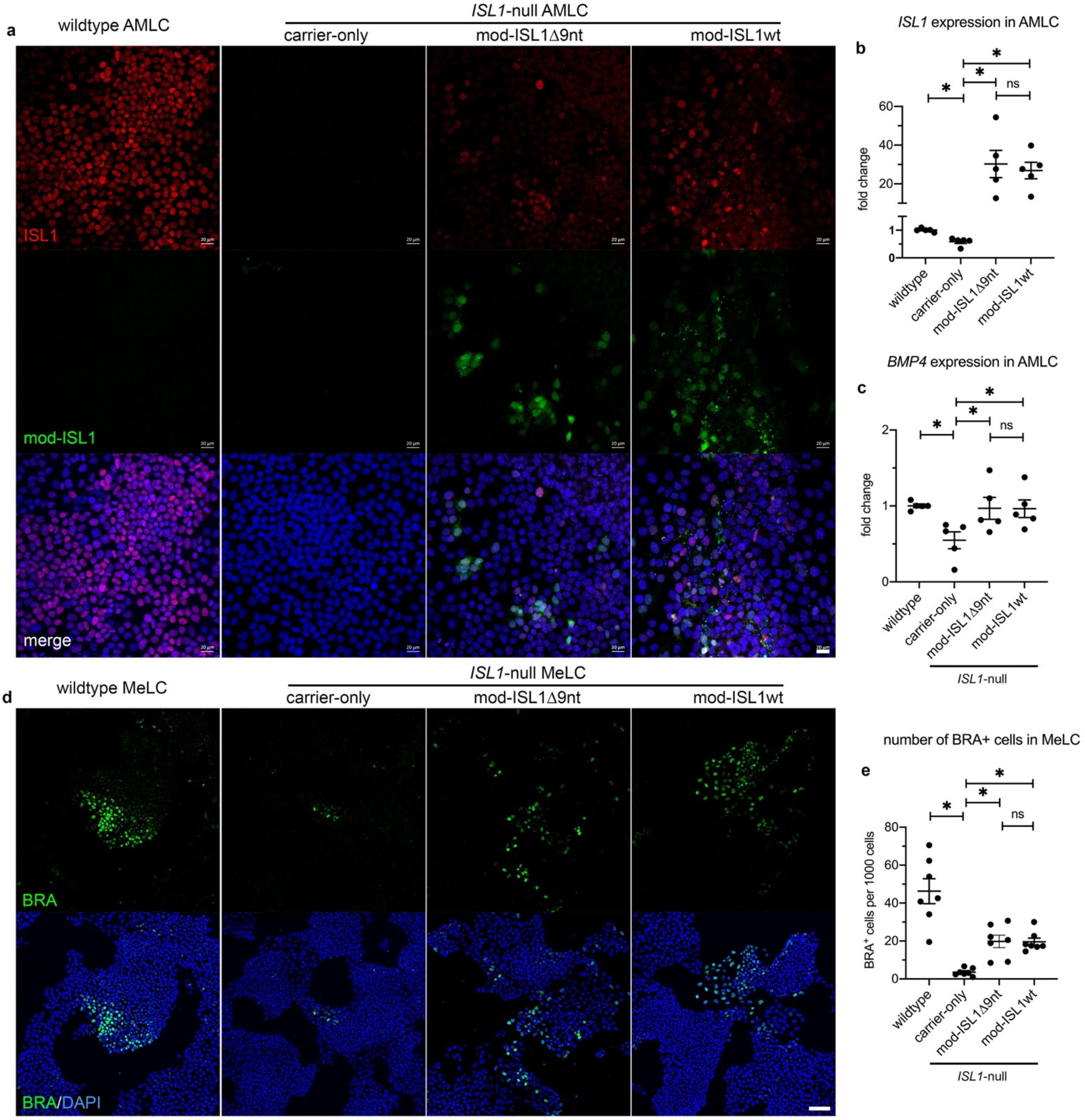


**Supplementary Fig. 11: Rescue of the phenotype in the transwell assay using *ISL1* modRNA**. Related to Fig. 5.

**a**, immunofluorescent staining for ISL1 (red) and imaging for GFP expressed by ISL1 modRNA (green) in wildtype AMLCs, as well as *ISL1*-null AMLCs. mod-ISL1Δ9nt: encodes ISL1 with a 3 amino acid deletion; mod-ISL1wt: encodes wildtype ISL1. Scale bar, 20 µm. n = 4 biologically independent experiments. **b,** *ISL1* expression in AMLCs after modRNA transfection as measured by qPCR using primers annealing to the 3' end. Data are presented as mean ± SEM and analyzed by two-tailed student's t-test, n = 5 biologically independent samples. * p-value < 0.05 (0.0034 for wildtype vs. carrier-only, 0.0133 for carrier-only vs. mod-ISL1Δ9nt and 0.0035 for carrier-only vs. modISL1wt). **c**, *BMP4* expression in AMLCs after modRNA transfection. Data are presented as mean ± SEM and analyzed by two-tailed student's t-test. n = 5 biologically independent samples. * p-value < 0.05 (0.0214 for wildtype vs. carrier-only, 0.0131 for carrier-only vs. mod-ISL1Δ9nt and 0.0154 for carrier-only vs. modISL1wt). **d**, immunofluorescence staining of BRA (green) in wildtype MeLCs, as well as *ISL1*-null MeLCs after transfection of the corresponding AMLCs with mutant (mod-ISL1Δ9nt) or wildtype (mod-ISL1wt) ISL1 modRNA. Scale bar 100 µm. **e**, quantification of BRA+ cells in (**d**). Data are presented as mean ± SEM and analyzed by two-tailed student's t-test. n = 7 biologically independent samples. * p-value < 0.05 (0.0003 for wildtype vs. carrier-only, 0.0045 for carrier-only vs. mod-ISL1Δ9nt and < 0.0001 for carrier-only vs. modISL1wt).

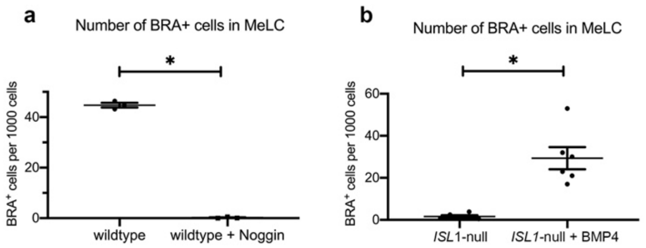


**Supplementary Fig. 12: Rescue of the phenotype in the transwell assay using BMP4.** Related to Fig. 5.

**a**, the number of BRA positive cells in wildtype MeLCs without and with Noggin treatment (quantitative analysis of Fig. 5f). Data are presented as mean ± SEM and analyzed by two-tailed student's t-test. n = 3 biologically independent samples. * p-value < 0.0001. **b**, the number of BRA positive cells in *ISL1*-null MeLCs without or with BMP4 treatment (quantitative analysis of Fig. 5g). Data are presented as mean ± SEM and analyzed by two-tailed student's t-test. n = 6 biologically independent samples. * p-value = 0.0004.

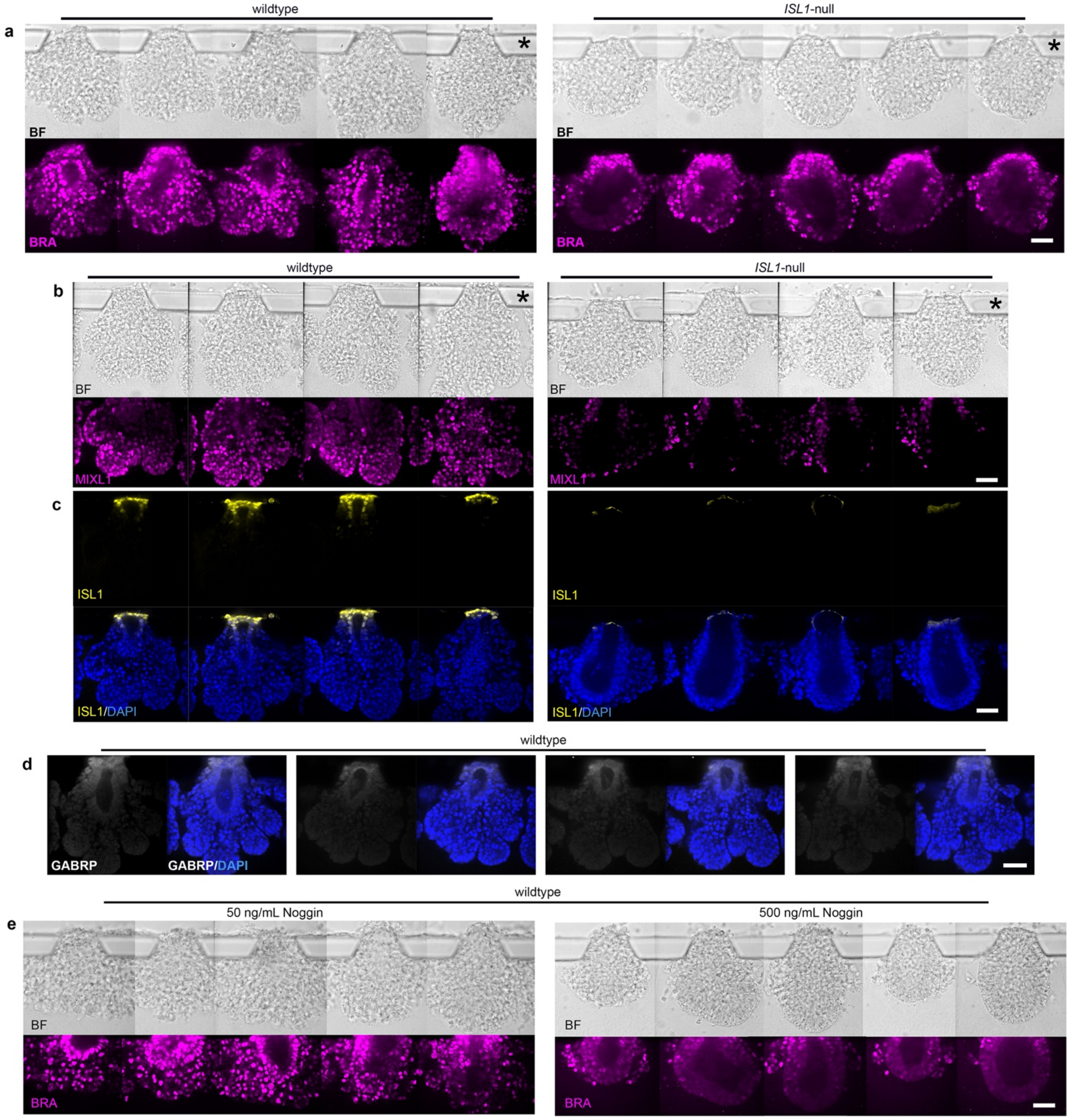


**Supplementary Fig. 13: the embryonic-like sac assay.** Related to Fig. 6.

**a**, Brightfield (BF) and immunofluorescent imaging for BRA in embryonic-like sacs derived from wildtype and *ISL1*-null hESCs. n = 30 each, scale bar, 50 µm. Asterisks indicate which embryonic-like sacs are presented in Fig. 6. **b**, Brightfield (BF) and immunofluorescent imaging for MIXL1 in embryonic-like sacs derived from wildtype and *ISL1*-null hESCs. n = 15 each, scale bar, 50 µm. Asterisks indicate which embryonic-like sacs are presented in Fig. 6. **c**, immunofluorescent imaging for ISL1 in embryonic-like sacs derived from wildtype and *ISL1*-null hESCs. Nuclei stained with DAPI (blue). n = 15 each, scale bar, 50 µm. **d**, immunofluorescent imaging for GABRP in embryonic-like sacs derived from wildtype hESCs. Nuclei stained with DAPI (blue). n = 15 each, scale bar, 50 µm. **e**, Brightfield (BF) and immunofluorescent imaging for BRA in wildtype hESCs derived embryonic-like sacs treated with low (50 ng/mL) or high (500 ng/mL) dose of Noggin. n = 15 each, scale bar, 50 µm.

**Description of Additional Supplementary Files**

**Supplementary Data 1: List of the most significant DEGs for each celltype in the wildtype NHP embryos.** Related to Fig. 1. P-values were calculated using a tow-tailed Welch's t-test. Unadjusted p-values as well as false discovery rates are reported.

**Supplementary Data 2: List of DEGs across pseudotime during naïve to primed transition.** Related to Supplementary Fig. 3. P-values were calculated using a two-sided Moran's I test. Unadjusted p-values as well as false discovery rates (q_values) are reported.

**Supplementary Data 3: List of all significant DEGs between AM-1 and AM-2 cells in the wildtype NHP embryos.** Related to Fig. 1 and Supplementary Fig. 5. P-values were calculated using a tow-tailed Welch's t-test. Unadjusted p-values as well as false discovery rates are reported.

**Supplementary Data 4: List of all members of the regulons highlighted in Fig. 2.** Related to Fig. 2 and Supplementary Fig. 6.

**Supplementary Data 5: List of all significant DEGs of mesodermal and amniotic cells between wildtype and mutant embryos.** Related to Fig. 4 and Supplementary Fig. 8. P-values were calculated using a tow-tailed Welch's t-test. Unadjusted p-values as well as false discovery rates are reported.

**Supplementary Data 6: List of chemicals, antibodies and primer sequences.**
