# Supplementary material for: Amnion signals are essential for mesoderm formation in primates
Source: Nat Commun. 2021 Aug 26;12:5126. doi: 10.1038/s41467-021-25186-2 (PMC8390679; doi:10.1038/s41467-021-25186-2)
Supplement: Supplementary file 8 — Reporting Summary [file 41467_2021_25186_MOESM8_ESM.pdf]

nature research

Corresponding author(s): Weizhi Ji, Fredrik Lanner, Yuyu Niu, Kenneth R. Chien

Last updated by author(s): Jul 3, 2021

Reporting Summary

Nature Research wishes to improve the reproducibility of the work that we publish. This form provides structure for consistency and transparency in reporting. For further information on Nature Research policies, see our Editorial Policies and the Editorial Policy Checklist.

Statistics

For all statistical analyses, confirm that the following items are present in the figure legend, table legend, main text, or Methods section.

n/a	Confirmed	
☐	☒	The exact sample size (n) for each experimental group/condition, given as a discrete number and unit of measurement
☐	☒	A statement on whether measurements were taken from distinct samples or whether the same sample was measured repeatedly
☐	☒	The statistical test(s) used AND whether they are one- or two-sided<br>*Only common tests should be described solely by name; describe more complex techniques in the Methods section.*
☒	☐	A description of all covariates tested
☐	☒	A description of any assumptions or corrections, such as tests of normality and adjustment for multiple comparisons
☐	☒	A full description of the statistical parameters including central tendency (e.g. means) or other basic estimates (e.g. regression coefficient) AND variation (e.g. standard deviation) or associated estimates of uncertainty (e.g. confidence intervals)
☐	☒	For null hypothesis testing, the test statistic (e.g. F, t, r) with confidence intervals, effect sizes, degrees of freedom and P value noted<br>*Give P values as exact values whenever suitable.*
☒	☐	For Bayesian analysis, information on the choice of priors and Markov chain Monte Carlo settings
☒	☐	For hierarchical and complex designs, identification of the appropriate level for tests and full reporting of outcomes
☒	☐	Estimates of effect sizes (e.g. Cohen's d, Pearson's r), indicating how they were calculated

Our web collection on statistics for biologists contains articles on many of the points above.

Software and code

Policy information about availability of computer code

Data collection	Single cell libraries were prepared using the 10x Genomics Chromium system and sequenced on an Illumina NovaSeq
Data analysis	All software used for the data analysis is described in detail in the methods section of the manuscript. The following software packages including version numbers were used:<br>R version 3.6.1; R Studio version 1.3; CellRanger Pipeline version 3.1.0; SoupX version 1.0.1; TrimGalore version 0.6.1; STAR version 2.5.3; featureCounts version 1.5.2; Seurat package version 3.1.3; SCTransform version 0.2.1; uwot version 0.1.5; Single Cell Signature Explorer version 3.1; Monocle3 version 0.2; SCENIC version 1.1.2-2; pySCENIC version 0.9.19; STRING-DB version 11.0; biomaRt version 2.40.5; Office 365 (Word, Excel); Adobe Creative Suite version 5.4.3.544; iMaris version 9.7; Zen 2012 SP1 version 8,1,5,484; Prism version 9.0.0 (86); Cas-OFFinder; SnapGene version 4.3.11; Infinity Analyze 6.3.0; Image Lab version 6.0.1 build 34 standard edition.

For manuscripts utilizing custom algorithms or software that are central to the research but not yet described in published literature, software must be made available to editors and reviewers. We strongly encourage code deposition in a community repository (e.g. GitHub). See the Nature Research guidelines for submitting code & software for further information.

Data

Policy information about availability of data

All manuscripts must include a data availability statement. This statement should provide the following information, where applicable:

- Accession codes, unique identifiers, or web links for publicly available datasets
- A list of figures that have associated raw data
- A description of any restrictions on data availability

The raw data, unfiltered count matrix and processed count matrix generated in this study have been deposited in the Gene Expression Omnibus (GEO) database under accession code GSE148683. The single cell RNA sequencing data from in vivo cynomolgus embryos used in this study are available in the GEO database under

accession code GSE74767. The single cell RNA sequencing data from the in vivo human embryo used in this study have not been deposited by the authors in a publicly available database yet, but the processed data is available through a web resource (http://www.human-gastrula.net/). Full-sized scans of western blots and full-sized immunofluorescent images are available in the source data file.

Field-specific reporting

Please select the one below that is the best fit for your research. If you are not sure, read the appropriate sections before making your selection.

☒ Life sciences ☐ Behavioural & social sciences ☐ Ecological, evolutionary & environmental sciences

For a reference copy of the document with all sections, see nature.com/documents/nr-reporting-summary-flat.pdf

Life sciences study design

All studies must disclose on these points even when the disclosure is negative.

Sample size	No sample-size calculations were performed. Instead, as many cells from the in vitro cultured embryos as technically possible were collected.
Data exclusions	Embryos that did not develop after thawing were excluded from the analysis. The exact numbers are reported in the manuscript. Data from the single-cell sequencing was filtered based on expression levels of mitochondrial genes and number of expressed genes. Details on the filtering cutoffs are provided in the supplementary data.
Replication	All experimental findings reported were reproducible. Each experiment was repeated independently for at least three times. Details on the number of replicates etc. are provided in the manuscript.
Randomization	Embryos from each batch were randomly allocated to being injected with CRISPR/gRNAs (ISL1 mutant) or to the wildtype group. For single cell sequencing all embryos from each batch were collected. For immunofluorescent imaging embryos were chosen randomly. In vitro experiments were randomized concerning well position, initiation of media change and start of treatments.
Blinding	For the imaging of the mutant embryos, blinding was not possible due to the obvious phenotype of the ISL1-mutant embryos. For the same reason blinding was not possible for specimen collection. Analysis of the single cell sequencing data did not require blinding, since it relies on objective, quantitative measurements including statistical testing, where appropriate. Concerning the in-vitro experiments of the transwell assays, the observer was blinded for the genotype of the corresponding picture during quantification of the immunofluorescent images. qPCR and western blots were carried out without blinding. All in vitro experiments concerning the embryonic-like sacs were performed by collaborators blinded to the corresponding genotype of the cells.

Reporting for specific materials, systems and methods

We require information from authors about some types of materials, experimental systems and methods used in many studies. Here, indicate whether each material, system or method listed is relevant to your study. If you are not sure if a list item applies to your research, read the appropriate section before selecting a response.

Materials & experimental systems

n/a	Involved in the study	
☐	☒	Antibodies
☐	☒	Eukaryotic cell lines
☒	☐	Palaeontology and archaeology
☐	☒	Animals and other organisms
☒	☐	Human research participants
☒	☐	Clinical data
☒	☐	Dual use research of concern

Methods

n/a	Involved in the study	
☒	☐	ChIP-seq
☒	☐	Flow cytometry
☒	☐	MRI-based neuroimaging

Antibodies

Antibodies used	anti-ISL1 (DSHB, 39.4D5, Lot 8/4/16), anti-ISL1 (Millipore, MABN1107, EP4182, Lot Q2473128), anti-OCT4 (DSHB, sc-5279, c-10, Lot D1813), anti-NANOG (R&D Systems, AF1997, Lot KKJ0619022), anti-Brachyury (R&D systems, AF2085, Lot KQP0619031), anti-MIXL1 (Sigma Aldrich, HPA005662, Lot 000009717), anti-GABRP (Thermo Fisher, PA5-46830, Lot WA3165881A), anti-SOX17 (R&D systems, AF1924, Lot KGA0916121), anti-GATA3 (Thermo Fisher, MA1-028, 1A12-1D9, Lot UD277690), anti-Vimentin (Millipore, AB5733, Lot 338039), anti-TFAP2C (Santa Cruz, sc-12762, 6E4/4, Lot B0620), Alexa Flour 488 donkey anti-goat IgG (Thermo Fisher, A11055, Lot 1737907),Alexa Flour 488 goat anti-mouse IgG1 (Thermo Fisher, A21121, Lot 2040297), Alexa Flour 488 goat anti-mouse IgG2b (Thermo Fisher, A21141, Lot 1832037), Alexa Flour 568 goat anti-mouse IgG2b (Thermo Fisher, A21144, Lot 1837737), Alexa Flour 568 goat anti-chicken IgG (Thermo Fisher, A11041, Lot 1691230), Alexa Flour 647 donkey anti-rabbit IgG (Thermo Fisher, A31573, Lot 1458609), anti-betaActin (Cell Signaling Technology, #5125, 13E5, Lot 6), HRP-linked anti-rabbit IgG (Cell Signaling Technology, #7074, Lot 26) and HRP-linked anti-mouse IgG (Cell Signaling Technology, #7076, Lot 32).
Validation	anti-OCT4, anti-NANOG, anti-Brachyury, anti-SOX17, anti-GATA3, and anti-TFAP2C are recommended by the manufactures for use in

Validation	immunofluorescence on human tissue and have been used in multiple publications in the field. In addition, they have been used in previous studies on primate samples (anti-OCT4 (Niu et. al., Science 2019), anti-NANOG (Niu et. al., Science 2019), anti-Brachyury (Ma et. al., Science 2019), anti-SOX17 (Niu et. al., Science 2019), anti-GATA3 (Kang et. al., Cell Reports 2018), anti-TFAP2C (Niu et. al., Science 2019)). anti-ISL1 (39.4D5) is recommend by the depositor for use in immunofluorescence on mouse samples and was used on human samples in multiple previous studies (Faye et. al., Brain Sci 2020; Mononen et. al., Stem Cells 2020). anti-MIXL1 was validated by the Human Protein Atlas for use in immunocytochemistry on human samples. anti-GABRP is recommended by the manufacturer for use in immunohistochemistry on human samples and has been used in previous publication (Engqvist et. al., BMC Cancer 2019). anti-Vimentin was validated by the manufacturer for use in immunocytochemistry on human tissue. anti-ISL1 (EP4182) is recommended by the manufacturer for use in western blot on human samples and has been used in previous publications (Zhang et. al., Theranostics 2019).

Eukaryotic cell lines

Policy information about cell lines

Cell line source(s)	HES-3, human embryonic stem cells and H9, human embryonic stem cells.
Authentication	Both cell lines (HES-3 and H9) were acquired directly from WiCell. Further authentication was not performed. However, genotyping for the ISL1-locus was carried out regularly to ensure correct allocation to groups.
Mycoplasma contamination	All cell lines were tested negative from mycoplasma contamination.
Commonly misidentified lines (See ICLAC register)	none

Animals and other organisms

Policy information about studies involving animals; ARRIVE guidelines recommended for reporting animal research

Laboratory animals	The cynomolgus macaque used in this study were all healthy female and aged 5-12 years old.
Wild animals	This study did not involve wild animals.
Field-collected samples	This study did not involve field-collected samples.
Ethics oversight	The ethics and all experimental protocols involved in this study were approved in advance by the Institutional Animal Care and Use Committee of LPBR in China (KBI K001115033/01,01) and by the Jordbruksverket in Sweden (ethical permit number N277/14).

Note that full information on the approval of the study protocol must also be provided in the manuscript.
