# Supplementary figures and images for: Amnion signals are essential for mesoderm formation in primates
Source: Nat Commun. 2021 Aug 26;12:5126. doi: 10.1038/s41467-021-25186-2 (PMC8390679; doi:10.1038/s41467-021-25186-2)

Fig. 2a

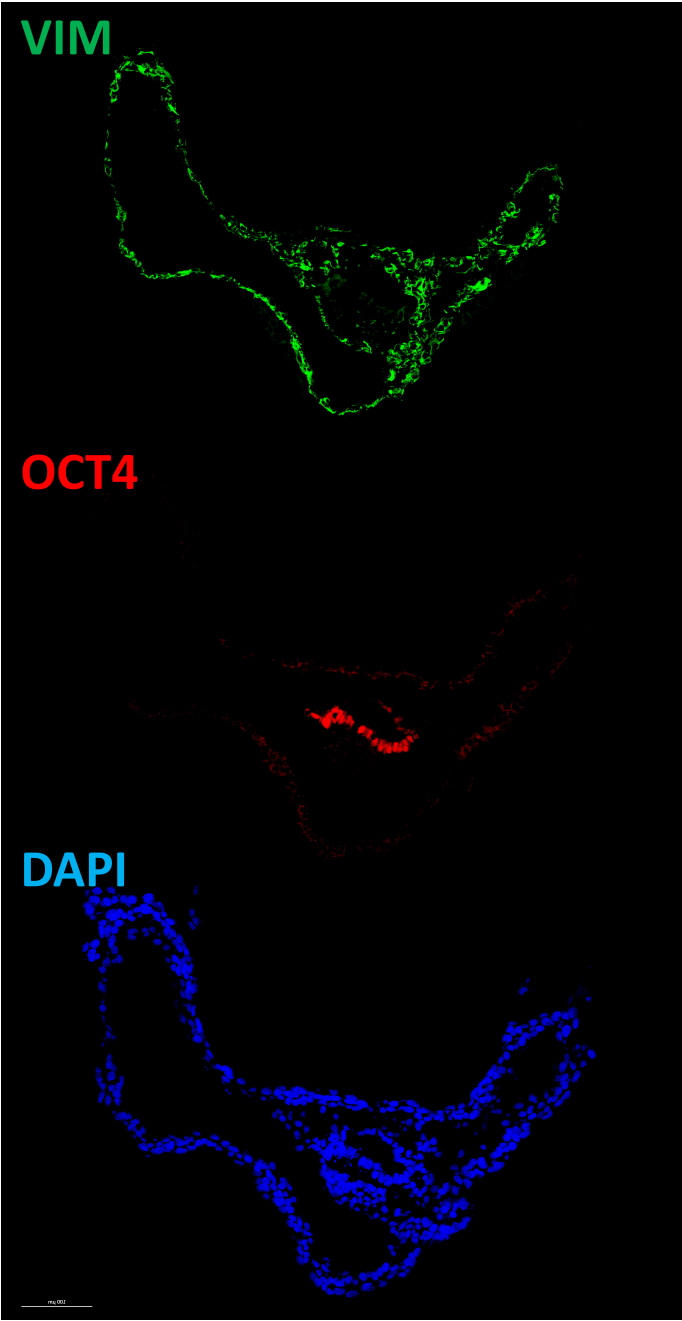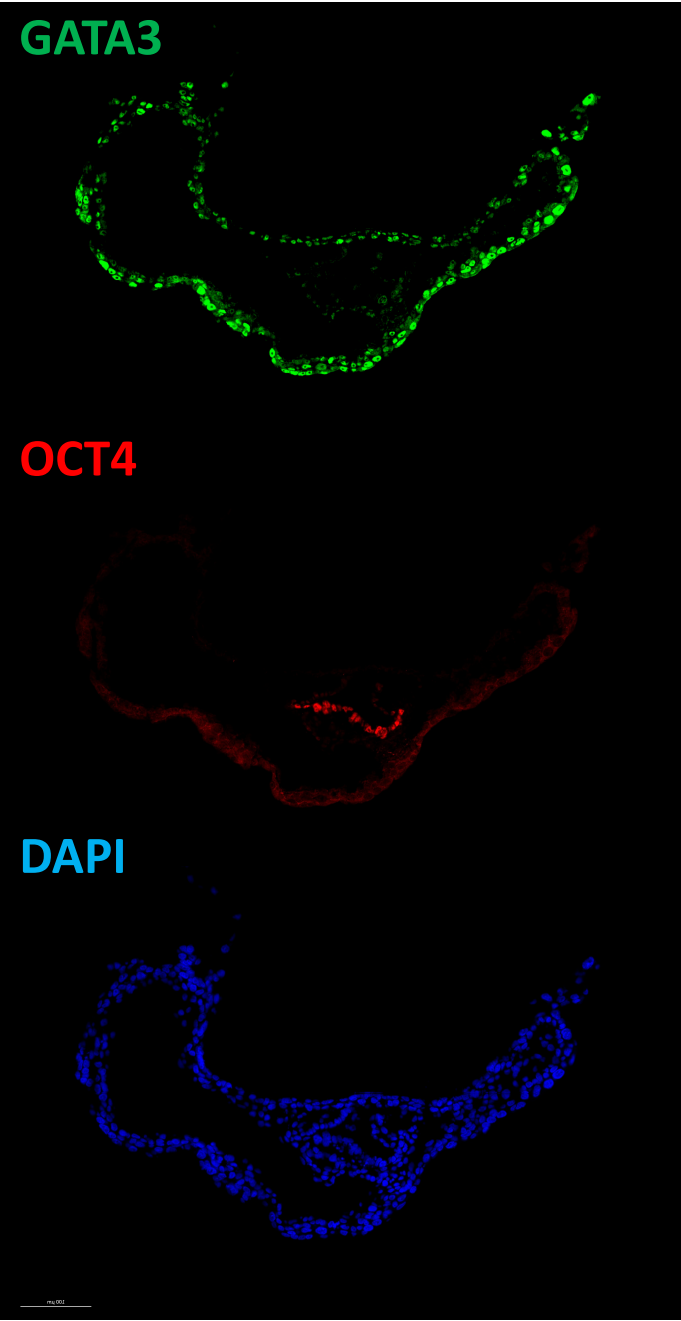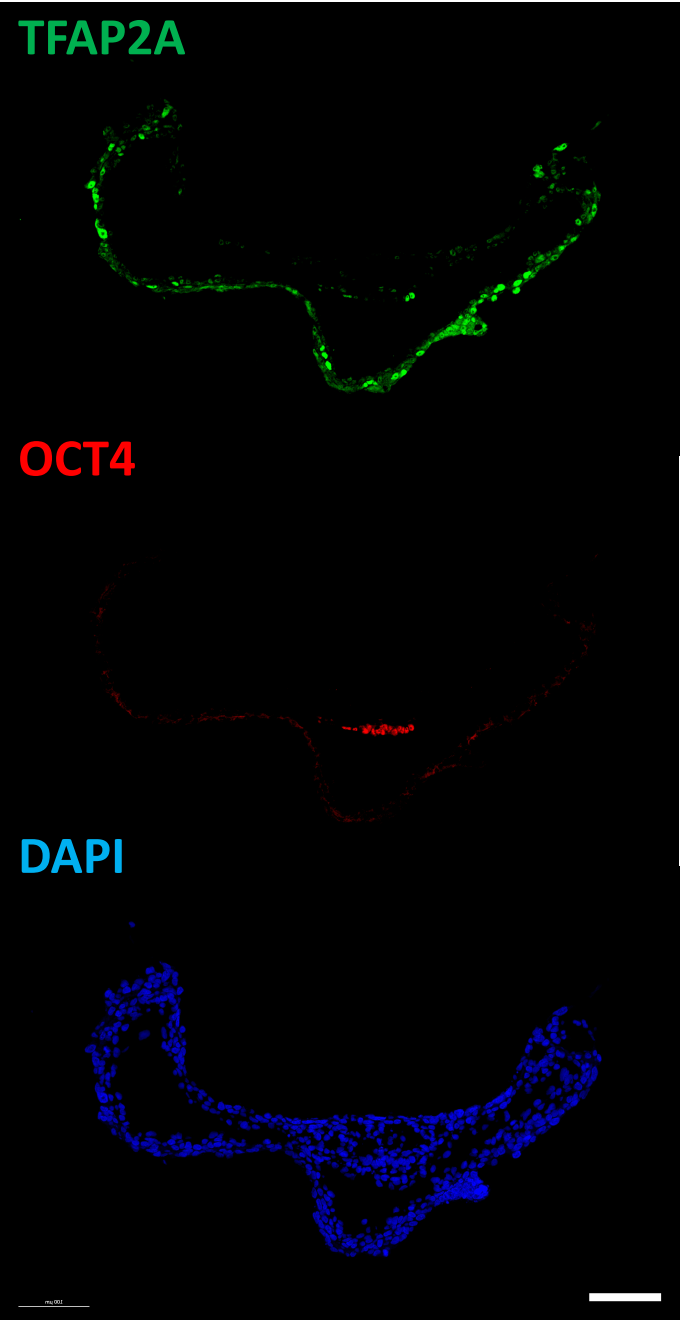

scale bar  
100um

Fig. 2b

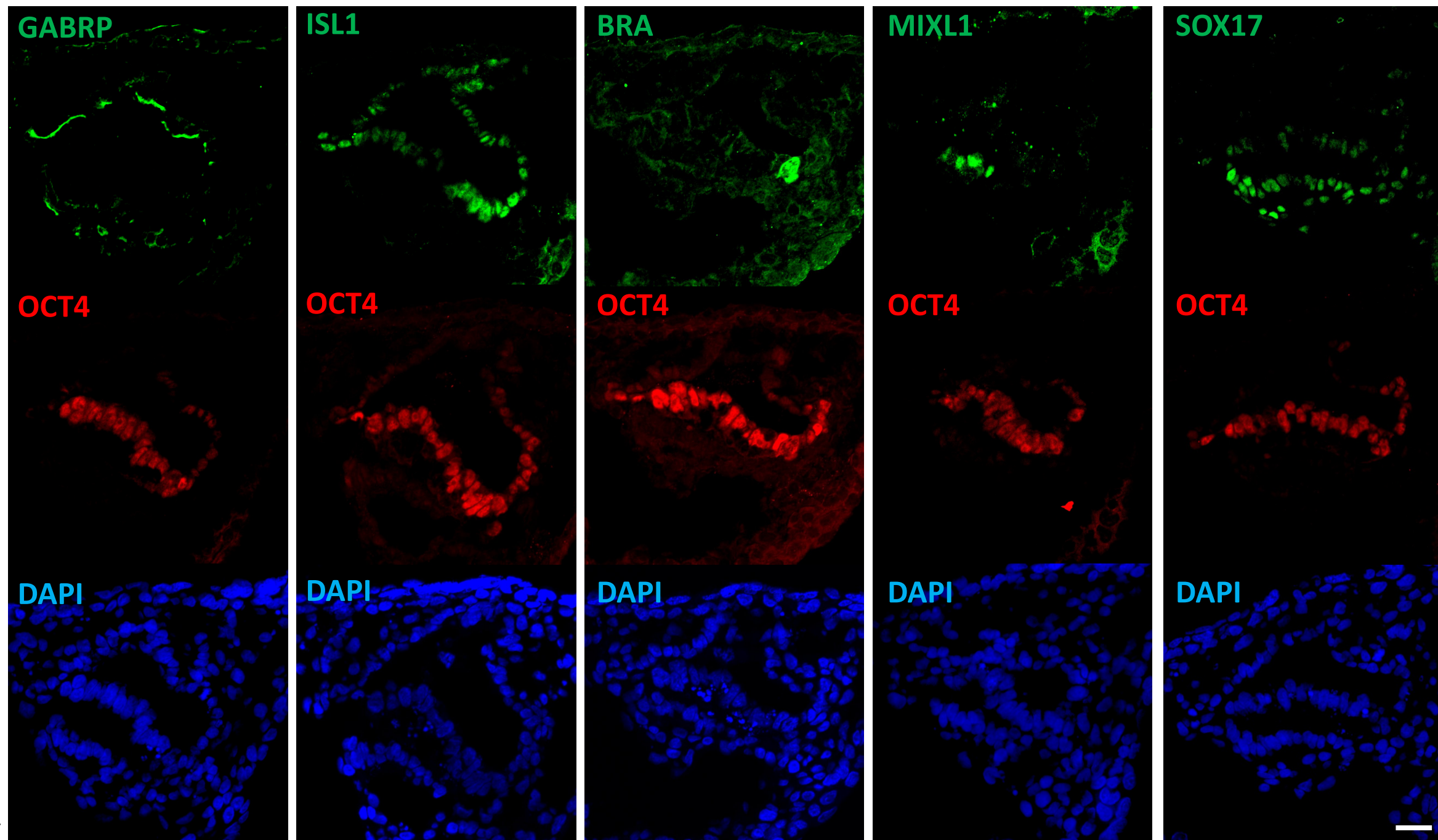

anti- $\beta$ -Actin

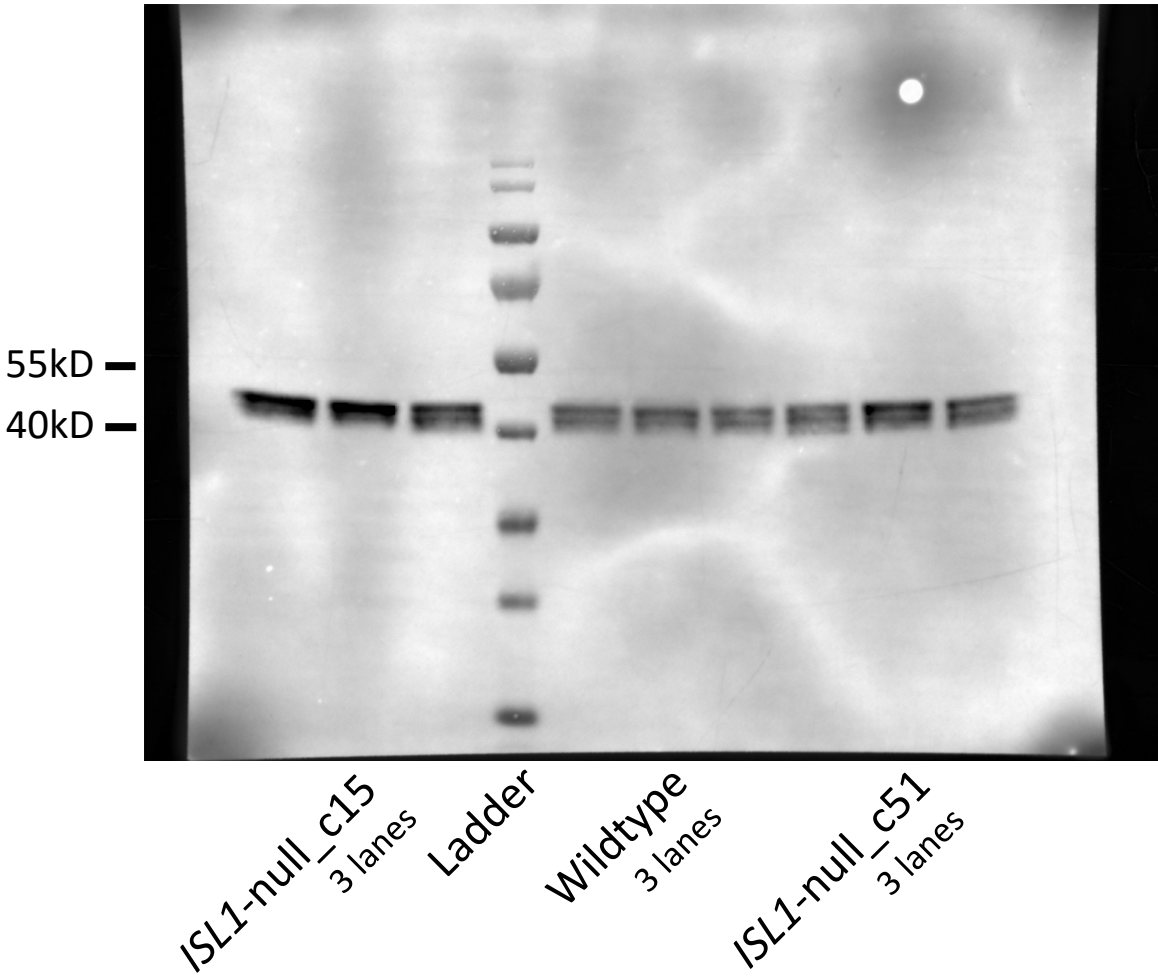

anti-ISL1

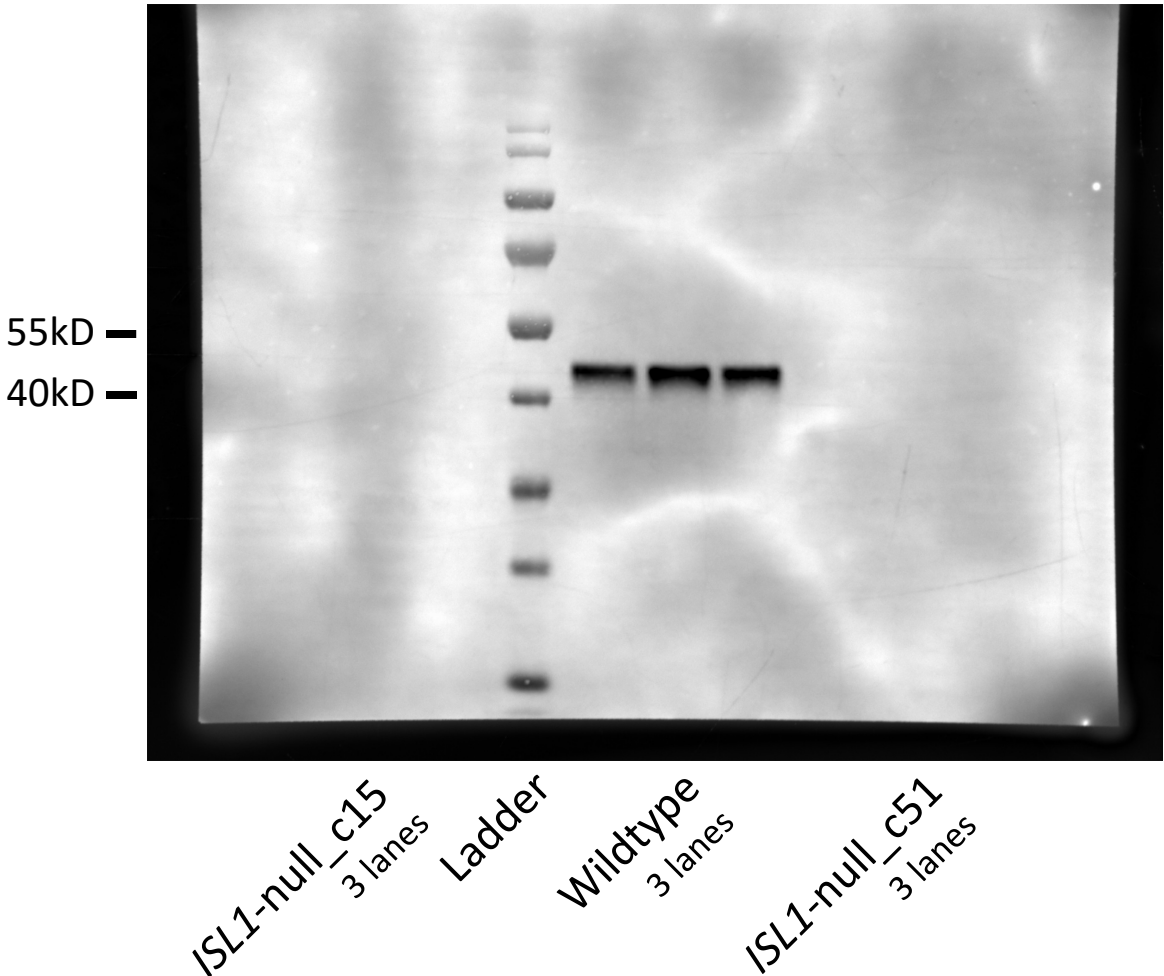

Supplement: Supplementary file 9 — source data [file 41467_2021_25186_MOESM9_ESM.zip › SourceDATA.pdf]
